# Nursing care in primary health care to tackle antimicrobial resistance in pregnant women with urinary tract infections

**DOI:** 10.1590/1980-220X-REEUSP-2025-0001en

**Published:** 2025-09-08

**Authors:** Jamile Leite de Figueiredo, Maria Clara Padoveze

**Affiliations:** 1 Universidade de São Paulo, Escola de Enfermagem, São Paulo, SP, Brazil.; 2 Einstein Hospital Israelita, São Paulo, SP, Brazil.

**Keywords:** Primary Health Care, Drug Resistance, Microbial, Pregnant People, Urinary Tract Infections, Family Health Nurses

## Abstract

**Objective:**

To identify pregnant women with urinary tract infections who are being monitored at a primary health care unit and their knowledge about antibiotics, as well as facilitating and challenging factors perceived by nurses that influence care, with a focus on antimicrobial resistance.

**Method:**

Exploratory, descriptive study with a quantitative approach, involving pregnant women with urinary tract infections undergoing antibiotic treatment at a municipal health unit in São Paulo and nurses working at the same location. Data were obtained from computerized systems, medical records, and interviews, and were synthesized and analyzed using Microsoft Excel and Stata software.

**Results:**

Of the 21 pregnant women included, 85.7% had knowledge about antibiotics. Of the 11 participating nurses, 36.4% did not identify the presence of facilitators, but 45.5% believed there were barriers to their care.

**Final considerations:**

Most pregnant women had basic knowledge about antibiotics and recognized the importance of guidance from nurses. Nurses identified barriers that point to the need for technical support. Developing a care booklet for nurses who care for pregnant women with urinary tract infections may be a promising strategy to optimize treatment and prevent antimicrobial resistance.

## INTRODUCTION

Urinary tract infections (UTIs) are inflammatory responses caused by the colonization and invasion of microorganisms in the urinary system, affecting approximately 150 million people globally. They are especially common during pregnancy, affecting 5% to 15% of pregnant women, mainly in the first trimester, with an overall prevalence of approximately 20%^(1,2,3)^. They can lead to maternal and fetal complications, and the various associated risk factors include health, socioeconomic, and demographic conditions. During pregnancy, other aggravating factors include anatomical, physiological, and hormonal changes, such as compression of the uterus and changes in hormone levels, which favor the occurrence of infections^(1)^.

At this stage, the most common clinical forms of UTIs are asymptomatic bacteriuria, followed by cystitis and pyelonephritis. The former is characterized by a urine culture result of more than 100,000 CFU/ml of the same bacteria in a midstream urine culture, without accompanying signs and symptoms. In other words, it refers to the presence of bacteria in the urine in large quantities, but without affecting the development of clinical manifestations^(1)^. Although it does not cause symptoms, its treatment is important in pregnant women, as it can lead to serious complications, such as progression to symptomatic UTIs, which can impact the health of the mother and fetus. There are other complications that may arise as a result of this progression, such as: pyelonephritis, thrombocytopenia, transient renal failure, intrauterine growth restriction, low birth weight, perinatal death, premature rupture of amniotic membranes, increased fetal mortality, prematurity, preterm labor, and febrile puerperium. In addition, it also increases the chance of postpartum endometriosis, eclampsia, anemia, and hypertension. Proper screening and treatment are essential to prevent these risks^(4)^.

In contrast, symptomatic UTI is accompanied by clinical signs and symptoms, i.e., the main difference between symptomatic UTI and asymptomatic bacteriuria lies in the presence of symptoms and the clinical definition of each condition^(2)^. Among symptomatic infections, cystitis occurs in the lower urinary tract, affecting the bladder and urethra, while pyelonephritis occurs in the upper urinary tract, when the kidneys are affected. Cystitis is the most common symptomatic form and can be complicated in people with health problems that increase the risk of treatment failure. Among the individuals most likely to experience serious complications are pregnant women, people who have undergone kidney transplants, those using indwelling urinary catheters, those with kidney disease, and those with concurrent diseases such as diabetes mellitus and immunosuppression^(4)^.

The possible occurrence of Antimicrobial Resistance (AMR) is an important factor to consider, as it makes the treatment of UTIs in pregnant women even more challenging. Currently, AMR is considered one of the main challenges in public health, resulting from the manifestation of resistance genes that compromise the effectiveness of antibiotics^(1)^, exacerbated by the inappropriate use of antibiotics, such as self- medication and dispensing without a prescription, some of the main causes of increased resistance rates^(1–4)^. Early diagnosis and appropriate treatment are essential to ensure the health of the pregnant woman and her baby, as an untreated UTI can lead to complications^(3)^.

In Brazil, with the aim of contributing to the fight against AMR, the National Health Surveillance Agency (Anvisa) has developed guidelines for the Antimicrobial Stewardship Program (ASP)^(5)^, a set of integrated actions that seek to ensure the responsible use of antimicrobials, with the participation of nurses in this process being vital^(6)^. The nursing team should monitor the administration of antimicrobials and educate patients on their proper use^(7,8)^.

In this scenario, Primary Health Care (PHC) plays a fundamental role, as it is responsible for meeting a high demand for infections, including UTIs, which are common during pregnancy. Nurses working in PHC play an essential role in educating about the responsible use of antimicrobials. Although a study indicated that 74.1% of them are unaware of the ASP, 92.9% consider the participation of nurses in these educational actions to be important^(9)^, and shared decision-making between professionals and users is also fundamental for the management of AMR. Among other activities, the role of nurses is crucial to ensure that bacteriological cultures are performed before the start of antibiotics and to monitor adequate adherence to treatment, avoiding the selection of resistant microorganisms^(7)^.

Prenatal care provided by nurses is essential to ensure the care of pregnant women, especially with regard to the management of urinary complaints. However, little is known about the nurse’s perspective on UTIs in pregnant women, that is, there is a scarcity of national studies exploring this topic, which suggests that nurses need to become more involved with the issue. In addition, workload can be a barrier to obtaining knowledge on the subject^(10)^.

Given this, this study aims to identify pregnant women with urinary tract infections being monitored at a Primary Health Care Unit (PHCU) and their knowledge about antibiotics, as well as facilitating factors and barriers perceived by nurses that influence care, with a focus on antimicrobial resistance. As part of the strategy to contribute to improving care, based on the findings, we intend to develop a booklet on care for pregnant women with UTIs for nurses in PHC, aiming to reduce AMR.

## METHOD

### Study Design

This is an exploratory, descriptive study with a quantitative approach.

### Setting

The study was conducted at the Parque Araribá PHCU, located in the Southern Coordination Office, under the Technical Health Supervision of Campo Limpo, in the municipality of São Paulo, which is currently administered by the Albert Einstein Israelite Hospital. The unit has ten Family Health Strategy (FHS) teams to serve approximately 36,000 users, which have a pre-established schedule, with daily slots for scheduled ongoing care and others for spontaneous access^(11)^. For scheduled care, such as prenatal care, appointments are divided according to a periodicity protocol: monthly until the 28^th^ week of pregnancy, biweekly from the 28^th^ to the 36^th^ week, weekly from the 36^th^ to the 40^th^ week, alternating between the nurse and the doctor^(12)^. This service provides approximately 107 prenatal consultations per month, according to data extracted from the Integrated Health Care Management System (SIGA Saúde) and Power BI – Indicator Management Portal of the unit in 2023.

### Population and Selection Criteria

Data collection was carried out between July 18 and November 7, 2024, after approval by the Research Ethics Committee, with the population consisting of pregnant women who were users of the PHCU and who received prescriptions for antibiotics for the treatment of UTIs. All pregnant women from the ten teams at the unit were considered preliminarily eligible, regardless of age group, age, gender, number of pregnancies, parity, number of children, race/skin color, number of partners, marital status, education, occupation, among others. The sample consisted of approximately 25 users, which corresponds to the maximum incidence rate of 20% reported in the literature^(13)^. The number was based on data from 2023, calculated from the literature and considering the average of 184 pregnant women registered at the PCC, as stated in the Mãe Paulistana Program, which is a program of the São Paulo City Hall to support pregnant women and newborns. The selection was made by analyzing the history of all medical records of pregnant women being monitored at the unit, evaluating both the physical medical records and the Electronic Citizen Medical Records (ECMR). After checking each medical record and identifying the pregnant women who had been prescribed antibiotics for the treatment of UTIs, the users were invited to participate. Nurses who provided care at the unit also participated in the study, with a sample of 11 professionals, including ten team nurses and one technical manager.

Regarding the inclusion and exclusion criteria, pregnant women with UTIs undergoing antibiotic treatment at the PHCU pregnant women with recurrent infections during pregnancy, and nurses working at the unit were included. Pregnant women from other PHCUs were excluded because there was insufficient data to complete the questionnaire. With regard to participating nurses, those who did not work at the PHCU during the data collection period due to vacation or leave were excluded. The first researcher in this study, who provided services at the unit, was also excluded.

### Data Collection

With regard to pregnant women, the sources of information were: (i) Power BI from the Primary Care and Care Networks Department of the Albert Einstein Israelite Hospital – a data analysis platform for generating reports and dashboards, developed by Microsoft, which allows users to view and transform data from various sources; (ii) SIGA Saúde platform – a digital platform for managing resources and services provided by the Municipal Health Secretariat of the City of São Paulo, which integrates the processes of health care, promotion, and regulation in municipal network establishments, supporting the Unified Health System (SUS); (iii) ECMR – national model for information management in PHC; (iv) medical records of pregnant women, available at the PHCU.

After identifying pregnant women with UTI, the researcher invited them to a face-to-face meeting, where she explained the study objectives and participation criteria. In cases where it was impossible for the pregnant woman to schedule a face-to- face appointment at the PHCU, the researcher conducted an active search through home visits. The nurses who provided prenatal care at the unit were invited to participate in the study to assess their knowledge of AMR and antibiotic use, as well as facilitating and challenging factors in care.

Data were obtained through specific questionnaires, one for pregnant women with UTI and another for nurses, using a five- point Likert scale (1-strongly disagree; 2-disagree; 3-neutral; 4-agree; 5-strongly agree) to measure the level of agreement. Its design was based on the authors’ experience, considering the routine care provided by nurses to pregnant women in PHCU services, and on the literature in the field. Experts who are members of the research group reviewed the instruments to ensure their comprehensiveness and applicability, which were submitted to a pilot stage, with adjustments made as necessary before their actual use for data collection. The questionnaires are available for consultation by contacting the corresponding author.

The questionnaire for pregnant women with UTI was organized into four parts: participant characterization – for personal data and patient profile; medical record data – filled in with medical record data, including gestational and obstetric data, information on acquired UTI, laboratory tests, and antibiotic prescriptions in the health history; patient-reported data – related to the pregnant woman’s complaints, as well as her symptoms and when the antibiotic was prescribed; knowledge about antibiotics – aimed at verifying the pregnant woman’s prior knowledge about antibiotics and how she carried out the treatment.

The nurse’s form was organized into two parts: nurse profile – containing information on length of service as a professional and in the FHS, improvements, and academic titles; nurse knowledge – including knowledge of antibiotic use, ADRs, and GPA.

### Data Analysis and Processing

The data collected (both from electronic records and questionnaires) were entered into a spreadsheet, using Microsoft Excel and Stata software as analysis tools. The results were analyzed using descriptive statistics, absolute and relative distribution of the variables studied and, when relevant, measures of central tendency and dispersion, with the support of a statistics professional. The data were presented in the form of graphs and tables, as appropriate.

### Ethical Aspects

The research was submitted to the Research Ethics Committee of the School of Nursing at the University of São Paulo, via Plataforma Brasil, in accordance with current legislation and with letters of authorization from the board and supervision of the Southern Coordination Office of the São Paulo City Hall. The data from the committee’s substantiated opinion are numbered 6,932,389 and CAAE 78707224.1.0000.5392.

## RESULTS

On August 1, 2024, after identifying pregnant women eligible for the study and applying the inclusion and exclusion criteria, invitations for in-person consultations were sent out by the PHCU teams. Initially, there was difficulty in getting patients to participate, as the teams were unable to meet the demand, with little time to contact them. The strategy was to place reminders in the medical records and send messages to medical professionals and nurses in the PHCU WhatsApp information group requesting referral for consultation with the researcher. Direct contact was also made with pregnant women through community health agents, which enabled data collection between September and October.

Initially, 29 participants were considered, with eight (27.6%) being excluded: two refusals, two not found, two moved out of the territory, two missed two consecutive appointments, and, after being offered a home visit, both refused. Of the 21 (72.4%) remaining eligible for detailed analysis, six underwent face-to-face consultations at the PCC and 15 received home visits. Table 1 shows the sociodemographic profile of pregnant women with UTI.

**Table 1:** Sociodemographic profile of pregnant women with urinary tract infections at the Parque Araribá Primary Health Care Unit – São Paulo, SP, Brazil, 2024. N = 21.

Sociodemographic variable	n	%
**Pregnancy**		
One	9	42.86
Two	9	42.86
Three or more	3	14.29
**Delivery**		
Normal delivery	7	33.33
Abortion	7	33.33
Hasn’t given birth yet	5	23.81
Cesarean section	2	9.52
**Number of living children**		
None	12	57.14
One	6	28.57
Two	1	4.76
Three	1	4.76
Five	1	4.76
Four	0	0.00
Number of partners*		
Only one	20	95.24
None	1	4.76
**Obstetric history**		
No	12	57.14
Yes	9	42.86
Abortion	7	33.33
Placental abruption	1	4.76
Gestational hypertension	1	4.76
**Gestational risk**		
Usual	17	80.95
High	4	19.05

Source: Prepared by the authors.

*At the time of the interview.

Of the pregnant women, almost half were primigravida and just under half were primiparous, while a minority had three or more previous pregnancies (multigravida). Regarding the type of delivery, just under half reported normal delivery, while a minority had a cesarean section; the rest had not yet experienced childbirth. Around one-third of pregnant women reported an episode of miscarriage, just over half had no live births, and most reported having only one partner. Table 2 presents the data from the UTI and other tests performed, with the respective results, also providing a description of the antibiotics prescribed.

**Table 2:** Clinical data related to urinary tract infection, tests performed with results, and description of prescribed antibiotics – São Paulo, SP, Brazil, 2024. N = 21.

Variable	n	%
**Urinary tract infection data**		
Confirmed by laboratory tests	9	42.86
Confirmed by symptoms and laboratory tests	8	38.10
Confirmed by symptoms	4	19.05
**Urine 1**		
Changed	19	90.48
No information	2	9.52
**Urine culture**		
Performed	18	85.71
Positive, *Escherichia coli*	10	47.62
Negative	5	23.81
Positive, no further information	1	4.76
Positive, mixed flora, suggestive of contamination	1	4.76
Positive, *Klebsiella pneumoniae*	1	4.76
No information	2	9.52
Not performed	1	4.76
**Antibiogram**		
Performed	10	47.62
Sensitive to nitrofurantoin, cephalexin, bactrim, nitrofurantoin, fosfomycin	3	28.58
Resistant to ciprofloxacin, norfloxacin, ampicillin, fosfomycin, trimethoprim, sulfamethoxazole	1	4.76
Not completed	9	42.86
No information	2	9.52
**Prescribed antibiotic**		
Cephalexin	10	47.62
Nitrofurantoin	8	38.10
Amoxicillin	1	4.76
Ceftriaxone	1	4.76
**Prescriber category**		
Family Health Strategy Doctor	16	76.19
Doctor from another service	4	19.05
Nurse	1	4.76
**Diagnosis**		
No information	12	57.14
Cystitis	2	9.52
Urinary tract infection of unspecified location	3	14.28
Asymptomatic bacteriuria	4	19.05
Other health issues*		
No	18	85.71
Yes	4	14.29
Diabetes	1	4.76
Anemia	1	4.76
Acute complaint	2	9.52
**Use of other medications during treatment**		
No	16	76.19
Yes	5	23.81
**Current complaints (after treatment)**		
No	18	85.71
Yes	3	14.29
**Improvement after treatment**		
Yes	18	85.71
No	3	14.29

Source: Prepared by the authors.

*Pregnant women may have more than one health problem.

Half of the pregnant women had UTIs confirmed exclusively by laboratory tests, while slightly less than half had confirmation based on both symptoms and tests. In most cases, the urine analysis showed abnormalities, with urine culture confirming the presence of *Escherichia coli* in concentrations above 100,000 CFU/mL in just over half of the cases, which is the main pathogen responsible for UTIs in pregnant women^(6)^. In our series, only one case had *Klebsiella pneumoniae* as the etiology. Despite its importance in guiding antimicrobial treatment, an antibiogram was not performed in the other half of the cases, which limited the possibility of targeted therapy. The data indicated that most pregnant women used antibiotics in the second trimester, followed by the first and third trimesters. Of the four pregnant women confirmed to have asymptomatic bacteriuria, two were in the first trimester of pregnancy (13 weeks and 9 weeks of gestation, respectively), identified in screening tests during the opening of prenatal care performed by the nurse. Regarding prophylaxis, cephalexin was mostly prescribed, followed by nitrofurantoin. The diagnosis was not detailed in most cases, and the physician was the main prescriber, most of whom were from the FHS. Only one nurse was identified as prescribing metronidazole cream. This finding may be due to a failure in the record, since the diagnosed indication is not compatible with the treatment.

Of the 21 pregnant women, five (23.8%) had a recurrence of UTI, with three of them (14.3%) having consulted with the FHS doctor and two (52.0%) with doctors from another service. In cases of recurrence, cefalexin was maintained in three cases (14.3%); in two (9.5%), fosfomycin was used. Regarding the time between prescriptions, the period of 7 to 14 days prevailed for the occurrence of the test reading and medication prescription, followed by 14 to 28 days. Most of those evaluated reported symptoms related to UTI, such as lower abdominal pain, dysuria, and pollakiuria, while the minority did not present significant symptoms, which may indicate underreporting of asymptomatic cases or cases with minor discomfort, possibly due to difficulties of pregnant women in expressing their symptoms. Only two did not adhere to treatment until the end, one of whom had asymptomatic bacteriuria. Table 3 presents data on pregnant women’s knowledge of antibiotics.

**Table 3:** Distribution of responses from pregnant women regarding knowledge of antibiotics – São Paulo, SP, Brazil, 2024. N = 21.

Question*	Completely disagree		Partially disagree		Neither disagree nor agree		Partially agree		Completely agree	
	n	%	n	%	n	%	n	%	n	%
Do you know what antibiotics are?	1	4.76	0	0.0	2	9.52	8	38.10	10	47.62
Have you ever used antibiotics without a prescription?	10	47.62	2	9.52	1	4.76	4	19.05	4	19.05
Do you usually have antibiotics at home?	7	33.33	5	23.81	1	4.76	4	19.05	4	19.05
Have you ever used leftover antibiotics at home?	12	57.14	2	9.52	4	19.05	1	4.76	2	9.52
Have you ever used antibiotics given to you by someone you know?	13	61.91	2	9.52	0	0.0	3	14.29	3	14.29
Do you know what prescription medications are for?	0	0.0	0	0.0	1	4.76	2	9.52	18	85.71
Have you ever forgotten to take your medication at the correct time during treatment?	10	47.62	1	4.76	6	28.6	0.0%	28.6	4	19.05
Did you ever stop taking your medication at any point during your treatment?	15	71.43	1	4.76	1	4.8	3	14.29	1	4.76
Did the professional who prescribed your medication advise you on how to take it correctly and why?	1	4.76	0	0.0	0	0.0	1	4.76	19	90.48
If you received guidance, were you satisfied with the information provided?	0	0.0	0	0.0	0	0.0	2	9.52	19	90.48
When you receive instructions on how to use antibiotics, do you usually follow them?	0	0.0	0	0.0	0	0.0	3	14.29	18	85.71
Do you consider it important to use antibiotics as instructed?	0	0.0	0	0.0	0	0.0	2	9.52	19	90.48
In your opinion, could the care provided in relation to instructions on antibiotic use be improved?	2	9.52	1	4.76	1	4.76	6	28.6	11	52.38
In your opinion, do nurses play an important role in providing care in relation to instructions on antibiotic use?	0	0.0	0	0.0	0	0.0	3	14.29	18	85.71
Do you consider it important for nurses to have a tool to better assist patients in the use of antibiotics?	1	4.76	0	0.0	0	0.0	3	14.3	17	80.95
Do you believe that this tool can improve care and, consequently, health?	0	0.0	0	0.0	0	0.0	4	19.05	17	80.95

Source: Prepared by the authors.

*All questions were answered by the pregnant women, with no missing responses.

Analysis of the questionnaire results revealed high agreement in responses to the question “Do you know what antibiotics are?”, indicating a good level of basic knowledge. Inappropriate use of antibiotics was rarely mentioned, although inappropriate practices were reported, such as the use of antibiotics without a prescription and leftover medications. Satisfaction with the guidance received was high, as was the level of adherence to the instructions provided by health professionals. Pregnant women highlighted the importance of nurses in providing guidance on the correct use of antibiotics.

Table 4 summarizes the results of the characterization of participating nurses.

**Table 4:** Profile of nurses – São Paulo, SP, Brazil, 2024. N = 11.

Variable	n	%
**Age**		
Between 40 and 60 years old	7	63.64
Up to 39 years old	3	27.27
Over 60 years old	1	9.09
**Length of service as a nurse in the Family Health Strategy**		
Between 10 and 15 years old	6	54.55
Up to 9 years old	3	27.27
Over 15 years old	2	18.18
**Always worked in Primary Health Care**		
Yes	8	72.73
No	3	27.27
**Type of undergraduate institution**		
Private	11	100.00
Other	0	0.0
**Postgraduate degree in Primary Health Care, public health, or collective health (completed)**		
Yes	9	81.82
No	2	18.18
**Postgraduate studies in progress in other areas**		
No	10	90.91
Yes	1	9.09

Source: Prepared by the authors.

With regard to length of service as a nurse, more than half of the professionals had between 10 and 15 years of experience, which demonstrates their technical maturity and accumulated expertise in clinical practice. Just under half reported up to 9 years of experience and more than 15 years. Regarding the length of service in the FHS, the data reflected a similar pattern, with just over half working between 10 and 15 years, while the other half was divided between up to 9 and more than 15 years of service. Most had always worked in PHC, demonstrating a solid professional trajectory in this field. Another relevant finding is that all had completed some postgraduate studies, especially in the area of collective health or public health. Regarding continuing education, with improvement in another area, only one (9.1%) reported pursuing another postgraduate degree. Another (9.1%) had completed residency in the area of practice. None were pursuing or had completed a doctorate. The assessment of nurses’ perceptions about antibiotics is shown in Table 5.

**Table 5:** Distribution of nurses’ responses regarding antibiotics, antimicrobial resistance, and the Antimicrobial Stewardship Program – São Paulo, SP, Brazil, 2024. N = 11.

Question*	Completely disagree		Partially disagree		Neither disagree nor agree		Partially agree		Completely agree	
	n	%	n	%	n	%	n	%	n	%
Do you know what antimicrobial resistance is?	1	9.09	0	0.0	0	0.0	2	18.2	8	72.73
Do you know what the Antimicrobial Stewardship Program is and what its purpose is?	3	27.3	2	18.2	1	9.09	4	36.36	1	9.09
Do you prescribe antibiotics established by nursing protocols from the municipality of São Paulo or institutional protocols?	5	45.46	0	0.0	0	0.0	2	18.2	4	27.27
Do you feel confident prescribing antibiotics?	4	36.36	1	9.09	1	9.09	2	18.2	3	27.27
Do you think you have enough knowledge to prescribe them?	3	27.27	1	9.09	2	18.2	3	27.3	2	18.2
Do you feel confident prescribing antibiotics to pregnant women with urinary tract infections?	4	36.36	1	9.09	2	18.2	2	18.2	2	18.2
In your practice, do you advise patients on the correct use of antibiotics and why?	0	0.0	0	0.0	0	0.0	2	18.2	9	81.82
Do you know what precautions should be advised to patients when they are prescribed antibiotics in order to reduce antimicrobial resistance?	0	0.0	0	0.0	0	0.0	4	36.36	7	63.64
Do you have knowledge about the management of antimicrobial resistance?	2	18.2	4	36.36	1	9.09	3	27.3	1	9.09
Have you received any training on antimicrobial resistance and its management?	6	54.55	1	9.09	2	18.2	1	9.09	1	9.09
In your opinion, do you think it is important to receive training that addresses antimicrobial resistance and its management in Primary Health Care?	0	0.0	0	0.0	0	0.0	0	0.0	11	100.0
Have you participated in educational activities related to antimicrobial resistance?	8	72.73	1	9.09	0	0.0	2	18.2	0	0.0
In your opinion, do you think it is important to have educational activities in Primary Health Care for the community that address antimicrobial resistance?	0	0.0	0	0.0	0	0.0	1	9.09	10	90.91
In your opinion, do you think it is important to have an Antimicrobial Stewardship Program in Primary Health Care?	0	0.0	0	0.0	1	9.09	1	9.09	9	81.82
Do you believe that nurses should be part of the Antimicrobial Stewardship Program in Primary Health Care to combat antimicrobial resistance?	0	0.0	0	0.0	1	9.09	1	9.09	9	81.82
Would you like to have a tool that can guide you in order to contribute to care regarding the use of antibiotics and management of antimicrobial resistance?	0	0.0	0	0.0	0	0.0	3	27.3	8	72.73
Do you believe that this tool can improve your care regarding the use of antibiotics?	0	0.0	0	0.0	0	0.0	2	18.2	9	81.82
Do you believe that this tool will contribute to reducing antimicrobial resistance in Primary Health Care?	0	0.0	0	0.0	1	9.09	1	9.09	9	81.82
In your opinion, are there barriers in your care that influence the fight against antimicrobial resistance?	0	0.0	0	0.0	3	27.3	3	27.3	5	45.46
In your opinion, are there facilitators in your care that influence the fight against antimicrobial resistance?	4	36.36	0	0.0	2	18.2	3	27.3	2	18.2

Source: Prepared by the authors.

*All questions were answered by nurses, with no missing responses.

The results showed that most professionals have a basic knowledge of AMR, with an acceptable level of responses to the question “Do you know what antimicrobial resistance is”. However, on the question “Do you know what the ASP is and its purpose?”, almost half of the nurses reported not knowing, while on the question “Have you ever received any training on AMR and its management?”, just over half said they had not. Almost half of the professionals also totally or partially disagreed that they felt safe prescribing antibiotics to pregnant women with UTIs (n = 5; 45.5%). As for the perception of the importance of training in AMR, all of them attributed a high degree of relevance to the issue, as well as the implementation of educational actions in PHC. With regard to the ASP, the majority felt that the program should be implemented in PHC, believing that nurses should be part of it in order to deal with AMR.

Among the nurses interviewed, five (45.5%) totally agreed with the existence of barriers in care that influence care in dealing with AMR. On the other hand, only two (18.2%) totally agreed with the existence of facilitators. With regard to the particularities of these barriers and facilitators, it was not possible to obtain more in-depth information due to the limitations of the method, which requires more detailed exploration in future studies.

## DISCUSSION

The sociodemographic profile of pregnant women with UTIs showed the influence of factors such as age, ethnicity, marital status, schooling and geographical location, which may be associated with inequalities in access to health care and impact the management and prevention of UTIs. This information is essential for the implementation of specific and effective interventions^(4,14)^, just as migration and unequal access to health services can contribute to the prevalence of UTIs in certain populations^(14)^.

The combination of urine 1, urine culture and antibiogram provides a complete and effective diagnostic approach for the management of UTI in pregnant women, allowing the etiological agents to be identified, antimicrobial sensitivity to be determined and treatment to be personalized. The use of tests such as urine culture and antibiogram to confirm the presence of pathogens and antimicrobial sensitivity is essential for choosing the right antibiotic. This approach not only improves clinical outcomes, but also reduces the incidence of AMR, especially in contexts where multidrug-resistant bacteria are common^(15)^. However, we found in our series that the antibiogram was not always carried out.

Another important factor is the timely performance of these laboratory tests, which are essential for the detection and confirmation of UTIs. Early diagnosis allows rapid interventions, reducing the risk of complications such as pyelonephritis and premature birth; studies indicate that delays in diagnosis are associated with greater morbidity in pregnant women and newborns^(16)^. Therefore, the time between diagnostic tests and the prescription of treatment is a determining factor in the clinical management of UTIs in pregnant women^(1)^, as it can have a direct impact on the progression of the infection and maternal-fetal outcomes, especially due to the physiological changes of pregnancy which increase susceptibility to complications^(17)^. On the other hand, antibiotics prescribed in relation to the time of pregnancy, the choice of type of antibiotic and the time of use should be carefully analyzed due to the possible impact on maternal and fetal health^(18)^. In this study, we found asymptomatic bacteriuria in the first trimester, which indicates the need for FHS nurses to pay attention to early infection screening.

With regard to drug treatment, the study showed appropriate management and the choice of antibiotics with low toxicity and risk for this group^(2,4)^. On the other hand, most of the diagnoses were undeclared, which can be attributed to the lack of adequate medical records. The lack of detailed information in the medical record can jeopardize early diagnosis and proper management of UTIs^(19)^. According to the results obtained, most of the prescribers are doctors from the FHS; this points to the need for greater interaction between the PHCU nurse and the prescriber to ensure more integrated care for pregnant women.

The use of questionnaires with a Likert scale has proved to be an effective tool for assessing the understanding and perceptions of pregnant women about UTIs, as it makes it possible to identify attitudes, beliefs and levels of knowledge in a standardized way, facilitating the analysis of qualitative and quantitative data. The results of this study showed that although there is a good level of knowledge and adherence to antibiotic use among pregnant women, inappropriate practices still occur^(20)^ and reflect gaps in understanding about the responsible use of antibiotics^(21)^. This knowledge plays a critical role in adhering to treatment and reducing UTI-related complications; in turn, suboptimal or inappropriate use of antibiotics can indicate failures in adherence to treatment. In addition, lack of adherence may be associated with socioeconomic barriers, lack of knowledge about the risks of untreated infections and hesitancy to use medication during pregnancy^(21,22)^. The role of nurses and the use of personalized educational tools are strategies to improve the management of antibiotic use and prevent ADR.

Satisfaction with the guidance received was high, as was the level of adherence to the instructions provided by health professionals; in fact, effective communication between health professionals and pregnant women plays a central role in treatment adherence. The pregnant women also highlighted the importance of the nurse’s role in providing guidance on the correct use of antibiotics, which reveals the need for educational tools to improve the care and health of users and the relevance of specific tools to reinforce clinical practice^(23)^.

With regard to length of time working as a nurse, more than half of the professionals had solid experience in PHC, which shows the technical maturity and expertise accumulated in clinical practice. This highlights the importance of continuous professional development strategies so that nurses are able to manage complex conditions such as UTI during pregnancy^(24)^.

With regard to length of time working in the FHS program, the data reflected a similar pattern, highlighting the importance of a solid professional career in this field. The experience accumulated in the FHS is a determining factor for the quality of care, since these professionals develop specific skills for PHC, including the ability to make early diagnoses and implement preventive interventions in the management of UTI. Continuing to work at this level of care, combined with specialization in public health, is a determining factor in the quality of care provided. This experience is essential, as it facilitates the development of specific skills. In addition, continuity in PHC allows for greater familiarity with care protocols and a greater bond with pregnant women^(25)^.

Another relevant fact is that all the nurses evaluated had completed postgraduate studies, mostly in the area of public health or collective health. This academic profile is a differentiator, considering that PHC requires professionals trained to address the social determinants of health, as well as promoting prevention and care strategies aimed at pregnant women^(25)^. This indicates that a significant number of participants were concerned with improving their skills in order to adapt effectively to the work environment, given that academic training and daily practice are intertwined in order to provide humanized and technically based care. It is expected that nurses with a specialization in public health will have a greater capacity to integrate technical- scientific knowledge into care practices, resulting in greater adherence by pregnant women to the proposed treatments^(26)^.

On the other hand, information regarding the assessment of health professionals’ perception of AMR and antibiotic management points to significant gaps in education and training. It is possible that the lack of continuing health education limits the role of nurses in this area^(27,28)^. On average, the professionals also showed insecurity in prescribing antibiotics to pregnant women with UTIs; however, high values were recorded for the perception of the importance of training in AMR, as well as the implementation of educational actions in PHC. A large proportion of nurses believe that it should be part of the ASP in PHC to deal with AMR. In this sense, there is room for training initiatives on how nurses should prescribe antibiotics for pregnant women with UTIs and the inclusion of institutional programs, such as the ASP, to strengthen the management of AMR^(9,10,29)^.

This study had some limitations, such as the small sample in a single PHCU, which did not allow for generalization; however, we would stress that this was an exploratory study which should be detailed in subsequent stages. Another limitation was the fact that the barriers and facilitators identified by the nurses were not studied in detail, which will be the subject of future studies.

## CONCLUSION

The research carried out at PCC Parque Araribá, in the municipality of São Paulo, brought to light relevant information that can contribute to strategies aimed at tackling AMR. Its results allow us to consider that the research’s guiding objectives were achieved, as it was possible to identify the prior knowledge about antibiotics of the pregnant women with UTI included in the study and the nurses’ perception of the existence of facilitators and challenges that influence care for this public.

We hope that the results will contribute to improving organizational, care and teaching practices, as well as stimulating reflections on public policies. We recommend the validation and implementation of educational technology with the target audience, through further research, in order to ensure the effectiveness of health education aimed at pregnant women with UTI in PHC.

Based on the findings, the next phase of this work will be the development of a care booklet for nurses who care for pregnant women with UTI, a promising strategy for optimizing treatment and preventing AMR, aligning care practices with the needs of the population served. The technical product in the form of a booklet will be made available on the website of the Public Policies, Epidemiology and Technologies in the Prevention of Healthcare-Related Infections research group (https://www.petiras.org/#).

This article is an excerpt from the master’s thesis entitled “The care of nurses in Primary Health Care to cope with antimicrobial resistance in pregnant women with urinary tract infection”, defended on April 16, 2025, in the Postgraduate Professional Master’s Program in Nursing in Primary Health Care in the Unified Health System (MPAPS) of the Nursing School of the University of São Paulo.

## Data Availability

The entire dataset supporting the results of this study is available upon request to the corresponding author, Jamile Leite de Figueiredo. The dataset is not publicly available because it contains information that compromises the privacy of the research participants.
