# Supplementary material for: Nursing care in primary health care to tackle antimicrobial resistance in pregnant women with urinary tract infections
Source: Rev Esc Enferm USP. 2025 Sep 8;59:e20250001. doi: 10.1590/1980-220X-REEUSP-2025-0001en (PMC12416863; doi:10.1590/1980-220X-REEUSP-2025-0001en)
Supplement: Translation

# Translation: O cuidado do enfermeiro na atenção primária à saúde para o enfrentamento da resistência antimicrobiana em gestantes com infecção urinária

**DOI:** 10.1590/1980-220X-REEUSP-2025-0001pt

**Authors:** Jamile Leite de Figueiredo, Maria Clara Padoveze

**Affiliations:** 1 Universidade de São Paulo, Escola de Enfermagem, São Paulo, SP, Brasil.; 2 Einstein Hospital Israelita, São Paulo, SP, Brasil.

**Keywords:** Atenção Primária à Saúde, Resistência Microbiana a Antibióticos, Gestante, Infecções Urinárias, Enfermeiros de Saúde da Família

## Abstract

**Objetivo:**

Identificar as gestantes com infecção do trato urinário em acompanhamento numa unidade básica de saúde e seu conhecimento sobre antibióticos, além de fatores facilitadores e desafiadores percebidos pelos enfermeiros que influenciam o cuidado, com foco na resistência antimicrobiana.

**Método:**

Estudo exploratório, descritivo, de abordagem quantitativa, com gestantes com infecção do trato urinário em tratamento com antibióticos em unidade do município de São Paulo e enfermeiros do mesmo local. Obtidos via sistemas informatizados, prontuário e entrevistas, os dados foram sintetizados e analisados com os *softwares Microsoft Excel* e *Stata*.

**Resultados:**

Das 21 gestantes incluídas, 85,7% tinham conhecimento sobre antibióticos. Dos 11 enfermeiros participantes, 36,4% não identificaram a presença de facilitadores, mas 45,5% acreditavam haver barreiras em seu atendimento.

**Considerações finais:**

A maioria das gestantes apresentou conhecimento básico sobre antibióticos e reconheceu a importância da orientação dos enfermeiros. Estes identificaram barreiras que apontam a necessidade de suporte técnico. Desenvolver uma cartilha de cuidado para enfermeiros que atendem a gestantes com infecção do trato urinário pode ser uma estratégia promissora para otimizar o tratamento e prevenir a resistência antimicrobiana.

## INTRODUÇÃO

As Infecções do Trato Urinário (ITU) são respostas inflamatórias causadas pela colonização e invasão de microrganismos no sistema urinário, afetando cerca de 150 milhões de pessoas globalmente. São especialmente comuns durante a gravidez, acometendo de 5% a 15% das gestantes, principalmente no primeiro trimestre, com prevalência geral de aproximadamente 20%^(1–3)^. Elas podem levar a complicações maternas e fetais e os diversos fatores de risco associados incluem condições de saúde, socioeconômicas e demográficas. Durante a gestação, outros agravantes incluem mudanças anatômicas, fisiológicas e hormonais, como a compressão do útero e as alterações nos níveis hormonais, que favorecem a ocorrência de infecções^(1)^.

Nessa fase, as formas clínicas das ITU mais comuns são a bacteriúria assintomática, seguida de cistite e pielonefrite. A primeira é caracterizada pelo resultado de urocultura de mais de 100.000 UFC/ml da mesma bactéria, em cultura de jato médio de urina, não acompanhada de sinais e sintomas. Em outras palavras, refere-se à presença de bactérias na urina em grande quantidade, mas não repercutindo no desenvolvimento de manifestações clínicas^(1)^. Embora não cause sintomas, seu tratamento é importante em gestantes, pois pode levar a complicações graves, como a evolução para ITU sintomáticas, que podem impactar a saúde da mãe e do feto. Há outras complicações que podem surgir em decorrência dessa evolução, como: pielonefrite, trombocitopenia, insuficiência renal transitória, limitação do crescimento intrauterino, baixo peso do recém-nascido (RN), óbito perinatal, ruptura prematura de membranas amnióticas, aumento da mortalidade fetal, prematuridade, trabalho de parto prematuro e puerpério febril. Além disso, também aumenta a chance de endometriose pós-parto, eclâmpsia, anemias e hipertensão. O rastreamento e tratamento adequados são essenciais para prevenir esses riscos^(4)^.

Em contraste, a ITU sintomática é acompanhada de sinais e sintomas clínicos, ou seja, a principal diferença entre ITU sintomática e bacteriúria assintomática reside na presença de sintomas e na definição clínica de cada condição^(2)^. Dentre as infecções sintomáticas, a cistite ocorre no trato urinário inferior, acometendo a bexiga e uretra, enquanto a pielonefrite ocorre no trato urinário superior, quando há acometimento renal. A cistite é a forma sintomática mais comum e pode complicar em pessoas com algum problema de saúde que aumente o risco de falha no tratamento. Entre os indivíduos com maior chance de evoluir com grande prejuízo estão as gestantes, as pessoas que passaram por transplante de rim, em uso de cateteres vesicais de demora, com patologias renais e com doenças simultâneas, como a diabetes mellitus e imunossuprimidas^(4)^.

A possível ocorrência de Resistência Antimicrobiana (RAM) é um fator importante a ser considerado, pois torna o tratamento das ITU em gestantes ainda mais desafiador. Atualmente, a RAM é considerada um dos principais desafios em saúde pública, resultante da manifestação de genes de resistência que comprometem a eficácia dos antibióticos^(1)^, sendo exacerbada pelo uso inadequado de antibióticos, como automedicação e dispensação sem prescrição, algumas das principais causas do aumento das taxas de resistência^(1–4)^. Seu diagnóstico precoce e tratamento adequado são fundamentais para garantir a saúde da gestante e do bebê, visto que uma ITU não tratada pode levar a complicações^(3)^.

No Brasil, visando a contribuir para o enfrentamento da RAM, a Agência Nacional de Vigilância Sanitária (Anvisa) desenvolveu diretrizes para o Programa de Gerenciamento de Antimicrobianos (PGA)^(5)^, um conjunto de ações integradas que busca garantir o uso responsável de antimicrobianos, sendo vital a participação de enfermeiros nesse processo^(6)^. A equipe de enfermagem deve monitorar a administração de antimicrobianos e educar os pacientes sobre seu uso adequado^(7,8)^.

Nesse cenário, a Atenção Primária à Saúde (APS) tem papel fundamental, pois é responsável por atender a uma alta demanda de infecções, incluindo as ITU, que são comuns durante a gestação. Os enfermeiros que atuam na APS desempenham papel essencial na educação sobre o uso responsável de antimicrobianos. Apesar de estudo ter indicado que 74,1% deles desconhecem o PGA, 92,9% consideram importante a participação da enfermagem nessas ações educativas^(9)^, também sendo fundamental a decisão compartilhada entre profissionais e usuários para a gestão da RAM. Entre outras atividades, a atuação dos enfermeiros é crucial para garantir que as culturas bacteriológicas sejam realizadas antes do início dos antibióticos e para monitorar a adequada adesão ao tratamento, evitando a seleção de microrganismos resistentes^(7)^.

O acompanhamento pré-natal realizado por enfermeiros é indispensável para garantir o cuidado de gestantes, especialmente no que diz respeito ao manejo de queixas urinárias, porém pouco se sabe sobre a perspectiva do enfermeiro em relação à ITU em gestantes, ou seja, existe escassez de estudos nacionais que exploram esse tema, o que sugere que os enfermeiros precisam se envolver mais com a questão. Ademais, a carga de trabalho pode ser uma barreira para obter conhecimento sobre o assunto^(10)^.

Diante disso, este estudo tem por objetivo identificar as gestantes com infecção do trato urinário em acompanhamento numa Unidade Básica de Saúde (UBS) e seu conhecimento sobre antibióticos, além de fatores facilitadores e barreiras percebidos pelos enfermeiros que influenciam o cuidado, com foco na resistência antimicrobiana. Como parte da estratégia para contribuir para a melhoria do cuidado, a partir dos achados, pretendemos desenvolver uma cartilha de cuidado a gestantes com ITU destinada a enfermeiros na APS, visando à redução da RAM.

## MÉTODO

### Desenho do Estudo

Trata-se de um estudo exploratório, descritivo, de abordagem quantitativa.

### Local

O estudo foi realizado na UBS Parque Araribá, localizada na Coordenadoria Sul, sob a Supervisão Técnica de Saúde do Campo Limpo, no município de São Paulo, cuja administração atualmente é realizada pelo Hospital Israelita Albert Einstein. A unidade conta com dez equipes da Estratégia Saúde da Família (ESF), para atendimento de cerca de 36 mil usuários, as quais possuem uma agenda preestabelecida, com vagas diárias de cuidado continuado programado e outras de acesso por demanda espontânea^(11)^. Para o atendimento programático, como no caso do pré-natal, as consultas são divididas conforme protocolo de periodicidade: mensais até à 28ª semana de gestação, quinzenais da 28ª à 36ª semana, semanal da 36ª à 40ª semana, alternadas entre o enfermeiro e o médico^(12)^. Nesse serviço, acontecem cerca 107 consultas de pré-natal por mês, segundo dados extraídos do Sistema Integrado de Gestão de Assistência à Saúde (SIGA Saúde) e Power BI – Portal Gestão de Indicadores da unidade em 2023.

### População e Critérios de Seleção

A coleta de dados foi realizada no período de 18 de julho a 7 de novembro de 2024, após aprovação do Comitê de Ética em Pesquisa, tendo gestantes usuárias da UBS que receberam prescrição de antibióticos para o tratamento de ITU como população. Foram consideradas preliminarmente elegíveis todas as gestantes das dez equipes da unidade, independentemente de faixa etária, idade, gênero, número de gestações, paridade, quantidade de filhos, raça/cor, número de parceiros, estado civil, escolaridade, ocupação, entre outros. A amostra foi composta por aproximadamente 25 usuárias, o que corresponde ao percentual máximo de 20% de incidência relatado na literatura^(13)^. O número baseou-se em dados de 2023, sendo calculado a partir da literatura e considerando a média de 184 gestantes cadastradas na UBS, conforme consta no Programa Mãe Paulistana, que é um programa da Prefeitura de São Paulo de apoio à gestante e ao recém-nascido. A seleção foi feita mediante análise do histórico de todos os prontuários de gestantes em acompanhamento na unidade, avaliando tanto o prontuário físico, quanto o Prontuário Eletrônico do Cidadão (PEC). Após a checagem de cada prontuário e a identificação das gestantes que haviam recebido prescrição de antibiótico para tratamento de ITU, as usuárias foram convidadas a participar. Também integraram o estudo enfermeiros que prestavam assistência na unidade, cuja amostra foi formada de 11 profissionais, sendo dez enfermeiros de equipe e um responsável técnico.

Quanto aos critérios de inclusão e exclusão, foram incluídas as gestantes com ITU em tratamento com antibióticos em atendimento na UBS, gestantes com infecções recorrentes durante o período da gestação e enfermeiros que atuavam na unidade. Foram excluídas as gestantes provenientes de outras UBS por não haver dados suficientes para o preenchimento do questionário. No que se refere aos enfermeiros participantes, foram excluídos os que não prestaram serviço na UBS no período da coleta por motivo de férias ou licença. A primeira pesquisadora deste estudo, prestadora de serviços na referida unidade, também foi excluída.

### Coleta de Dados

Com relação às gestantes, as fontes de informação foram: (i) Power BI da Diretoria de Atenção Primária e Redes Assistenciais do Hospital Israelita Albert Einstein – plataforma de análise de dados para geração de relatórios e *dashboards*, desenvolvida pela Microsoft, que permite que os usuários visualizem e transformem dados de diversas fontes; (ii) Plataforma SIGA Saúde – plataforma digital de gestão dos recursos e serviços prestados pela Secretaria Municipal da Saúde da Prefeitura de São Paulo, que integra os processos de assistência, promoção e regulação da saúde nos estabelecimentos da rede municipal, apoiando o Sistema Único de Saúde (SUS); (iii) PEC – modelo nacional de gestão da informação na APS; (iv) prontuário das gestantes, disponível na UBS.

Após a identificação de gestantes com ITU, a pesquisadora convidou-as para um encontro presencial, no qual explicou os objetivos do estudo e os critérios de participação. Nos casos de impossibilidade da gestante para agendamento de consulta presencial na UBS, a pesquisadora realizou a busca ativa por meio de visita domiciliar. Os enfermeiros que realizavam o acompanhamento de pré-natal na unidade foram convidados a participar do estudo para verificar seu conhecimento sobre RAM e uso de antibióticos, assim como fatores facilitadores e desafiadores presentes no cuidado.

Os dados foram obtidos por meio de questionários específicos, sendo um para as gestantes com ITU e outro para os enfermeiros, utilizando escala Likert de cinco pontos (1-discordo totalmente; 2-discordo parcialmente; 3-neutro; 4-concordo parcialmente; 5-concordo totalmente) para mensuração do nível de concordância. Sua elaboração baseou-se na experiência das autoras, considerando a rotina da assistência prestada pelos enfermeiros a gestantes nos serviços de APS, e em bibliografia da área. Especialistas membros do grupo de pesquisa revisaram os instrumentos para assegurar sua abrangência e aplicabilidade, os quais foram submetidos a uma etapa-piloto, com ajustes realizados conforme a necessidade antes do uso efetivo para coleta de dados. Os questionários estão disponíveis para consulta por meio de contato com a autora de correspondência.

O formulário de gestante com ITU foi organizado em quatro partes: caracterização da participante – destinada aos dados pessoais e perfil da paciente; dados do prontuário – preenchida com os dados de prontuário, incluindo dados gestacionais e obstétricos, informações sobre a ITU adquirida, exames laboratoriais e prescrição de antibióticos constante do histórico de saúde; dados referidos do paciente – relativo às queixas da gestante, assim como seus sintomas e momento em que foi prescrito o antibiótico; conhecimento sobre os antibióticos – voltada a verificar o conhecimento prévio da gestante sobre os antibióticos e como realizou o tratamento.

O formulário do enfermeiro foi organizado em duas partes: perfil do enfermeiro – constando informações sobre o tempo de atuação como profissional e na ESF, aprimoramentos e títulos acadêmicos; conhecimento do enfermeiro – incluindo o conhecimento do uso dos antibióticos, RAM e PGA.

### Análise e Tratamento dos Dados

Os dados coletados (tanto dos registros eletrônicos quanto dos questionários) foram inseridos em planilha, utilizando como ferramenta de análise os softwares *Microsoft Excel* e *Stata*. Os resultados foram analisados por meio de estatística descritiva, distribuição absoluta e relativa das variáveis estudadas e, quando pertinente, medida de tendência central e dispersão, com o apoio de profissional de Estatística. Os dados foram apresentados em forma de gráficos e tabelas, conforme a pertinência.

### Aspectos Éticos

A pesquisa foi submetida ao Comitê de Ética em Pesquisa da Escola de Enfermagem da Universidade de São Paulo, via Plataforma Brasil, conforme as legislações vigentes e mediante cartas de autorização da diretoria e supervisão da Coordenadoria Sul da Prefeitura Municipal de São Paulo. Os dados do parecer consubstanciado do comitê têm a numeração 6.932.389 e CAAE 78707224.1.0000.5392.

## RESULTADOS

Em 1º de agosto de 2024, após o levantamento das gestantes elegíveis para o estudo e a aplicação dos critérios de inclusão e exclusão, fez-se o convite para consulta presencial por meio das equipes da UBS. No início, houve dificuldade de adesão das pacientes, uma vez que as equipes não conseguiam atender à demanda, com tempo curto para contato com elas. A estratégia foi colocar lembretes nos prontuários e enviar mensagens aos profissionais médicos e enfermeiros no grupo informativo de WhatsApp da UBS solicitando o encaminhamento para consulta com a pesquisadora. Também foi realizado contato direto com as gestantes por intermédio das agentes comunitárias de saúde, o que viabilizou a coleta de dados entre setembro e outubro.

Foram consideradas inicialmente 29 participantes, sendo excluídas oito (27,6%): duas recusas, duas não encontradas, duas mudaram do território, duas faltaram em dois agendamentos seguidos e, após ofertada a visita domiciliar, ambas recusaram. Das 21 (72,4%) restantes aptas para análise detalhada, seis passaram por consultas presenciais na UBS e 15 receberam visitas domiciliares. A Tabela 1 mostra o perfil sociodemográfico das gestantes com ITU.

**Tabela 1:** Perfil sociodemográfico das gestantes com infecção do trato urinário da Unidade Básica de Saúde Parque Araribá – São Paulo, SP, Brasil, 2024. N = 21.

Variável sociodemográfica	n	%
**Gestação**		
Uma	9	42,86
Duas	9	42,86
Três ou mais	3	14,29
**Paridade**		
Parto normal	7	33,33
Aborto	7	33,33
Não teve parto ainda	5	23,81
Parto cesárea	2	9,52
**Número de filhos vivos**		
Nenhum	12	57,14
Um	6	28,57
Dois	1	4,76
Três	1	4,76
Cinco	1	4,76
Quatro	0	0,00
Número de parceiros*		
Somente um	20	95,24
Nenhum	1	4,76
**Antecedentes obstétricos**		
Não	12	57,14
Sim	9	42,86
Aborto	7	33,33
Deslocamento de placenta	1	4,76
Hipertensão gestacional	1	4,76
**Risco gestacional**		
Habitual	17	80,95
Alto	4	19,05

Fonte: Elaborado pelas autoras.

*No momento da entrevista.

Das gestantes, quase a metade era primigesta e pouco menos da metade, primípara, enquanto a minoria tinha três ou mais gestações anteriores (multigesta). Sobre o tipo de parto, pouco menos da metade relatou parto normal, enquanto uma minoria teve parto cesárea; as demais ainda não tiveram experiência de parto. Em torno de um terço das gestantes relatou episódio de aborto, pouco mais da metade não possuía filhos nascidos vivos e a maioria relatou ter apenas um parceiro. A Tabela 2 apresenta os dados da ITU e demais exames realizados, com os respectivos resultados, trazendo também a descrição dos antibióticos prescritos.

**Tabela 2:** Dados clínicos referentes à infecção do trato urinário, exames realizados com resultados e descrição dos antibióticos prescritos – São Paulo, SP, Brasil, 2024. N = 21.

Variável	n	%
**Dados da infecção do trato urinário**		
Confirmada por exames laboratoriais	9	42,86
Confirmada por sintomas e exames laboratoriais	8	38,10
Confirmada por sintomas	4	19,05
**Urina 1**		
Alterado	19	90,48
Sem informação	2	9,52
**Urocultura**		
Realizado	18	85,71
Positiva, *Escherichia coli*	10	47,62
Negativa	5	23,81
Positiva, sem outras informações	1	4,76
Positiva, flora mista, sugestiva de contaminação	1	4,76
Positiva, *Klebsiella pneumoniae*	1	4,76
Sem informação	2	9,52
Não realizado	1	4,76
**Antibiograma**		
Realizado	10	47,62
Sensível a nitrofurantoina, cefalexina, bactrim, nitrofurantoina, fosfomicina	3	28,58
Resistência à ciprofloxacina, norfloxacina, ampicilina, fosfomicina, trimetoprim, sulfametoxazol	1	4,76
Não realizado	9	42,86
Sem informação	2	9,52
**Antibiótico prescrito**		
Cefalexina	10	47,62
Nitrofurantoina	8	38,10
Amoxicilina	1	4,76
Ceftriaxona	1	4,76
**Categoria do prescritor**		
Médico da Estratégia Saúde da Família	16	76,19
Médico de outro serviço	4	19,05
Enfermeiro	1	4,76
**Diagnóstico**		
Sem informação	12	57,14
Cistite	2	9,52
Infecção do trato urinário de localização não especificada	3	14,28
Bacteriúria assintomática	4	19,05
Outros problemas de saúde*		
Não	18	85,71
Sim	4	14,29
Diabetes	1	4,76
Anemia	1	4,76
Queixa aguda	2	9,52
**Uso de outro medicamento durante o tratamento**		
Não	16	76,19
Sim	5	23,81
**Queixas atuais (após o tratamento)**		
Não	18	85,71
Sim	3	14,29
**Melhora após o tratamento**		
Sim	18	85,71
Não	3	14,29

Fonte: Elaborado pelas autoras.

*Gestantes podem apresentar mais do que um problema de saúde.

Metade das gestantes teve a ITU confirmada exclusivamente por exames laboratoriais, enquanto pouco menos da metade apresentou confirmação tanto por sintomas quanto por exames. Na maioria dos casos, a análise sumária da urina mostrou-se alterada, tendo a urocultura confirmado a presença de *Escherichia coli* em concentrações superiores a 100.000 UFC/mL em pouco mais da metade dos casos, sendo esse o principal patógeno responsável pelas ITU em gestantes^(6)^. Em nossa casuística, apenas um caso teve etiologia por *Klebsiella pneumoniae.* Apesar de sua importância para guiar o tratamento antimicrobiano, o antibiograma não foi realizado na outra metade dos casos, o que limitou a possibilidade de terapêutica dirigida. Os dados indicaram que a maior parte das gestantes utilizou antibióticos no segundo trimestre, seguido pelo primeiro e terceiro trimestres. Das quatro gestantes confirmadas para bacteriúria assintomática, duas estavam no primeiro trimestre gestacional (13 semanas e 9 semanas de gestação, respectivamente), sendo identificada nos exames de rastreio durante a abertura do pré-natal realizada pelo enfermeiro. Com relação à profilaxia, em sua maioria foi prescrita a cefalexina, seguida da nitrofurantoina. O diagnóstico não foi detalhado na maioria dos casos e o médico foi o principal prescritor, a maior parte proveniente da ESF. Apenas um enfermeiro foi identificado como prescritor de metronizadol creme. Este achado pode ser devido a uma falha no registro, uma vez que a indicação diagnosticada não está compatível com o tratamento.

Das 21 gestantes, cinco (23,8%) tiveram recidiva da ITU, tendo três delas (14,3%) passado por consulta com o médico da ESF e duas (52,0%), com médicos de outro serviço. Na recidiva, foi mantida a escolha pela cefalexina em três casos (14,3%); em dois (9,5%), utilizou-se fosfomicina. Em relação ao tempo entre as prescrições, prevaleceu o período de 7 a 14 dias para a ocorrência da leitura do exame e prescrição da medicação, seguido de 14 a 28 dias. A maioria das avaliadas relatou sintomas relacionados à ITU, como dor no baixo ventre, disúria e polaciúria, enquanto a minoria não apresentou sintomas significativos, o que pode indicar subnotificação dos casos assintomáticos ou com desconfortos menores, possivelmente por dificuldades das gestantes de expressar os sintomas. Apenas duas não aderiram ao tratamento até o final, uma das quais teve bacteriúria assintomática. A Tabela 3 apresenta os dados relativos ao conhecimento das gestantes sobre antibióticos.

**Tabela 3:** Distribuição das respostas das gestantes sobre o conhecimento de antibióticos – São Paulo, SP, Brasil, 2024. N = 21.

Questão*	Discordo totalmente		Discordo parcialmente		Neutro		Concordo parcialmente		Concordo totalmente	
	n	%	n	%	n	%	n	%	n	%
Sabe o que é antibiótico?	1	4,76	0	0,0	2	9,52	8	38,10	10	47,62
Já fez uso de antibiótico sem receita?	10	47,62	2	9,52	1	4,76	4	19,05	4	19,05
Costuma ter antibióticos em casa?	7	33,33	5	23,81	1	4,76	4	19,05	4	19,05
Já fez uso dos antibióticos que sobraram em casa?	12	57,14	2	9,52	4	19,05	1	4,76	2	9,52
Já fez uso de antibióticos dados por algum conhecido?	13	61,91	2	9,52	0	0,0	3	14,29	3	14,29
Sabe para que são os medicamentos receitados?	0	0,0	0	0,0	1	4,76	2	9,52	18	85,71
Esqueceu-se, em algum momento do tratamento, de tomar o remédio no horário correto?	10	47,62	1	4,76	6	28,6	0,0%	28,6	4	19,05
Em algum momento do tratamento, interrompeu o uso da medicação?	15	71,43	1	4,76	1	4,8	3	14,29	1	4,76
Foi orientado pelo profissional quando lhe passou a medicação sobre o uso correto dela e o porquê?	1	4,76	0	0,0	0	0,0	1	4,76	19	90,48
Se recebeu orientação, ficou satisfeito com a informação dada?	0	0,0	0	0,0	0	0,0	2	9,52	19	90,48
Quando recebe a orientação do uso, costuma utilizar conforme foi orientado?	0	0,0	0	0,0	0	0,0	3	14,29	18	85,71
Considera importante o uso do antibiótico conforme orientado?	0	0,0	0	0,0	0	0,0	2	9,52	19	90,48
Na sua opinião, o atendimento com relação às orientações dadas sobre o uso do antibiótico pode melhorar?	2	9,52	1	4,76	1	4,76	6	28,6	11	52,38
Na sua opinião, o enfermeiro tem uma atuação importante no atendimento com relação às orientações dadas sobre o uso do antibiótico?	0	0,0	0	0,0	0	0,0	3	14,29	18	85,71
Considera importante que o profissional enfermeiro tenha um instrumento voltado a auxiliar melhor os pacientes no uso dos antibióticos?	1	4,76	0	0,0	0	0,0	3	14,3	17	80,95
Acredita que esse instrumento pode melhorar o atendimento e, consequentemente, a saúde?	0	0,0	0	0,0	0	0,0	4	19,05	17	80,95

Fonte: Elaborado pelas autoras.

*Todas as questões foram respondidas pelas gestantes, não havendo perdas de respostas.

A análise dos resultados do questionário revelou alta concordância em respostas para a questão “Sabe o que é antibiótico?”, indicando bom nível de conhecimento básico. A utilização inadequada de antibióticos foi pouco citada, embora tenham sido relatadas práticas inadequadas, como uso de antibióticos sem receita e de medicamentos sobrantes. A satisfação com as orientações recebidas foi elevada, assim como o nível de adesão às instruções fornecidas pelos profissionais de saúde. As gestantes destacaram a importância da atuação do enfermeiro na orientação sobre o uso correto dos antibióticos.

A Tabela 4 reúne os resultados da caracterização dos enfermeiros participantes.

**Tabela 4:** Perfil dos enfermeiros – São Paulo, SP, Brasil, 2024. N = 11.

Variável	n	%
**Idade**		
Entre 40 e 60 anos	7	63,64
Até 39 anos	3	27,27
Acima de 60 anos	1	9,09
**Tempo de atuação como enfermeiro da Estratégia Saúde da Família**		
Entre 10 e 15 anos	6	54,55
Até 9 anos	3	27,27
Acima de 15 anos	2	18,18
**Sempre atuou na Atenção Primária à Saúde**		
Sim	8	72,73
Não	3	27,27
**Tipo de instituição de graduação**		
Particular	11	100,00
Outros	0	0,0
**Pós-graduação na área de Atenção Primária à Saúde, saúde pública ou saúde coletiva (concluída)**		
Sim	9	81,82
Não	2	18,18
**Pós-graduação em andamento em outras áreas**		
Não	10	90,91
Sim	1	9,09

Fonte: Elaborado pelas autoras.

No que diz respeito ao tempo de atuação como enfermeiro, mais da metade dos profissionais tinha entre 10 e 15 anos de experiência, o que evidencia a maturidade técnica e a *expertise* acumulada na prática clínica. Pouco menos da metade informou até 9 anos de atuação e mais de 15 anos. Em relação ao tempo de atuação na ESF, os dados refletiram um padrão semelhante, com pouco mais da metade atuando entre 10 e 15 anos, enquanto a outra metade dividiu-se entre até 9 e mais de 15 anos de atuação. A maioria sempre atuou na APS, evidenciando uma trajetória profissional sólida nesse âmbito. Outro dado relevante é que todos haviam concluído alguma pós-graduação, em especial, na área de saúde coletiva ou saúde pública. A respeito da continuidade no ensino, com aperfeiçoamento em outra área, apenas um (9,1%) informou cursar outra pós-graduação. Outro (9,1%) havia concluído a residência na área de atuação. Nenhum estava cursando ou havia concluído o doutorado. A avaliação da percepção dos enfermeiros acerca dos antibióticos está demonstrada no Tabela 5.

**Tabela 5:** Distribuição das respostas dos enfermeiros acerca dos antibióticos, a resistência antimicrobiana e o Programa de Gerenciamento de Antimicrobianos – São Paulo, SP, Brasil, 2024. N = 11.

Questão*	Discordo totalmente		Discordo parcialmente		Neutro		Concordo parcialmente		Concordo totalmente	
	n	%	n	%	n	%	n	%	n	%
Sabe o que é a resistência antimicrobiana?	1	9,09	0	0,0	0	0,0	2	18,2	8	72,73
Sabe o que é o Programa de Gerenciamento de Antimicrobianos e o seu objetivo?	3	27,3	2	18,2	1	9,09	4	36,36	1	9,09
Prescreve antibióticos estabelecidos por protocolos de enfermagem do município de São Paulo ou protocolos institucionais?	5	45,46	0	0,0	0	0,0	2	18,2	4	27,27
Sente-se seguro para prescrever antibióticos?	4	36,36	1	9,09	1	9,09	2	18,2	3	27,27
Acha que tem conhecimento suficiente para prescrevê-los?	3	27,27	1	9,09	2	18,2	3	27,3	2	18,2
Sente-se seguro para prescrever antibióticos para gestantes com infecção do trato urinário?	4	36,36	1	9,09	2	18,2	2	18,2	2	18,2
Em seus atendimentos, orienta o paciente sobre uso correto do antibiótico e o porquê?	0	0,0	0	0,0	0	0,0	2	18,2	9	81,82
Sabe quais são os cuidados que devem ser orientados para o paciente quando ele recebe a prescrição de um antibiótico a fim de reduzir a resistência antimicrobiana?	0	0,0	0	0,0	0	0,0	4	36,36	7	63,64
Tem conhecimento sobre o manejo da resistência antimicrobiana?	2	18,2	4	36,36	1	9,09	3	27,3	1	9,09
Já recebeu algum treinamento com relação à resistência antimicrobiana e seu manejo?	6	54,55	1	9,09	2	18,2	1	9,09	1	9,09
Na sua opinião, acha importante receber treinamentos que abordem a resistência antimicrobiana e seu manejo na Atenção Primária à Saúde?	0	0,0	0	0,0	0	0,0	0	0,0	11	100,0
Já participou de ações educativas com relação à resistência antimicrobiana?	8	72,73	1	9,09	0	0,0	2	18,2	0	0,0
Na sua opinião, acha importante ter ações educativas na Atenção Primária à Saúde para a comunidade que abordem a resistência antimicrobiana?	0	0,0	0	0,0	0	0,0	1	9,09	10	90,91
Na sua opinião, acha que é importante ter o Programa de Gerenciamento de Antimicrobianos na Atenção Primária à Saúde?	0	0,0	0	0,0	1	9,09	1	9,09	9	81,82
Acredita que o enfermeiro deve fazer parte do Programa de Gerenciamento de Antimicrobianos na Atenção Primária à Saúde para o enfrentamento da resistência antimicrobiana?	0	0,0	0	0,0	1	9,09	1	9,09	9	81,82
Gostaria de ter um instrumento que possa guiá-lo a fim de contribuir no cuidado com relação ao uso de antibióticos e manejo da resistência antimicrobiana?	0	0,0	0	0,0	0	0,0	3	27,3	8	72,73
Acredita que esse instrumento pode melhorar seu atendimento com relação ao uso de antibióticos?	0	0,0	0	0,0	0	0,0	2	18,2	9	81,82
Acredita que esse instrumento irá contribuir para redução da resistência antimicrobiana na Atenção Primária à Saúde?	0	0,0	0	0,0	1	9,09	1	9,09	9	81,82
Em sua opinião, existem barreiras no seu atendimento que influenciam o cuidado no enfrentamento da resistência antimicrobiana?	0	0,0	0	0,0	3	27,3	3	27,3	5	45,46
Em sua opinião, existem facilitadores no seu atendimento que influenciam o cuidado no enfrentamento da resistência antimicrobiana?	4	36,36	0	0,0	2	18,2	3	27,3	2	18,2

Fonte: Elaborado pelas autoras.

*Todas as questões foram respondidas pelos enfermeiros, não havendo perdas de respostas.

Os resultados mostraram que a maioria dos profissionais tem conhecimento básico sobre RAM, com nível aceitável de respostas para a questão “Sabe o que é a resistência antimicrobiana”. Contudo, na pergunta “Sabe o que é o PGA e seu objetivo”, quase a metade dos enfermeiros informou não saber, enquanto, na questão “Já recebeu algum treinamento com relação à RAM e seu manejo”, pouco mais da metade afirmou que não. Quase a metade dos profissionais também discordam totalmente ou parcialmente que se sentem seguros para prescrever antibióticos para gestantes com ITU (n = 5; 45.5%). Já quanto à percepção da importância do treinamento em RAM, todos atribuíram alto grau de relevância à questão, assim como da implementação de ações educativas na APS. Com relação ao PGA, a maioria considerou que o programa deve ser implantado na APS, acreditando que o enfermeiro deve fazer parte dele nesse âmbito para o enfrentamento da RAM.

Entre os enfermeiros entrevistados, cinco (45,5%) concordavam totalmente na existência de barreiras no atendimento que influenciam o cuidado no enfrentamento da RAM. Em contrapartida, apenas dois (18,2%) concordaram totalmente com a existência de facilitadores nesse atendimento. No que tange às particularidades dessas barreiras e facilitadores, não foi possível obter essas informações mais aprofundadas pela limitação do método, o que requer uma exploração mais detalhada em estudos futuros.

## DISCUSSÃO

O perfil sociodemográfico das gestantes com ITU evidenciou a influência de fatores como idade, etnia, estado civil, escolaridade e localização geográfica, que podem estar associados a desigualdades no acesso ao cuidado de saúde e impactar o manejo e a prevenção de ITU. Essas informações são essenciais para a implementação de intervenções específicas e eficazes^(4,14)^, assim como a migração e acesso desigual aos serviços de saúde podem contribuir para a prevalência de ITU em determinadas populações^(14)^.

A combinação de urina 1, urocultura e antibiograma proporciona uma abordagem diagnóstica completa e eficaz para a gestão de ITU em gestantes, permitindo identificar os agentes etiológicos, determinar a sensibilidade aos antimicrobianos e personalizar o tratamento. O uso de exames como urocultura e antibiograma para confirmar a presença de patógenos e a sensibilidade antimicrobiana é essencial para a escolha do antibiótico correto. Essa abordagem não apenas melhora os desfechos clínicos, mas também reduz a incidência de RAM, especialmente nos contextos em que bactérias multirresistentes são comuns^(15)^. Contudo, verificamos em nossa casuística que nem sempre o antibiograma foi realizado.

Outro fator importante é a realização oportuna desses exames laboratoriais, pois é fundamental para a detecção e a confirmação das ITU. Diagnósticos precoces permitem intervenções rápidas, reduzindo o risco de complicações, como pielonefrite e parto prematuro; estudos indicam que atrasos no diagnóstico estão associados a maior morbidade em gestantes e neonatos^(16)^. Portanto, o tempo entre a realização de exames diagnósticos e a prescrição do tratamento é fator determinante no manejo clínico das ITU em gestantes^(1)^, pois pode impactar diretamente a progressão da infecção e os desfechos materno-fetais, especialmente devido às alterações fisiológicas da gestação que aumentam a suscetibilidade a complicações^(17)^. Já os antibióticos prescritos correlacionados ao momento gestacional, à escolha do tipo de antibiótico e ao momento a ser utilizado devem ser cuidadosamente analisados devido aos possíveis impactos sobre a saúde materno-fetal^(18)^. Neste estudo, verificamos a ocorrência de bacteriúria assintomática no primeiro trimestre, o que indica a necessidade de atenção do enfermeiro da ESF para o rastreio precoce de infecção.

Com relação ao tratamento medicamentoso, o estudo demonstrou o manejo adequado e a escolha de antibiótico de baixa toxicidade e risco para esse grupo^(2,4)^. Já o diagnóstico, em sua maioria, não foi declarado, o que pode ser atribuído à falta de registro adequado no prontuário. A ausência de informações detalhadas no histórico médico pode comprometer o diagnóstico precoce e o manejo adequado das ITU^(19)^. De acordo com os resultados obtidos, o prescritor em sua maioria é da categoria médica, proveniente da ESF; isso aponta a necessidade de maior interação do enfermeiro da UBS com o profissional prescritor para assegurar um cuidado mais integrado para a gestante.

A aplicação de questionários com escala Likert tem se mostrado uma ferramenta eficaz para avaliar o entendimento e as percepções de gestantes sobre ITU, pois possibilita identificar de forma padronizada atitudes, crenças e níveis de conhecimento, facilitando a análise de dados qualitativos e quantitativos. Os resultados deste estudo demonstraram que, embora haja um bom nível de conhecimento e adesão ao uso de antibióticos entre as gestantes, práticas inadequadas ainda ocorrem^(20)^ e refletem lacunas no entendimento sobre o uso responsável de antibióticos^(21)^. Esse conhecimento desempenha papel crítico na adesão ao tratamento e na redução de complicações relacionadas à ITU; por sua vez, o uso subótimo ou inadequado de antibióticos pode indicar falhas na adesão ao tratamento. Além disso, a falta de adesão pode estar associada a barreiras socioeconômicas, falta de conhecimento sobre os riscos de infecções não tratadas e hesitação em utilizar medicamentos durante a gravidez^(21,22)^. A atuação dos enfermeiros e o uso de instrumentos educativos personalizados são estratégias para melhorar a gestão do uso de antibióticos e prevenir a RAM.

A satisfação com as orientações recebidas foi alta, assim como o nível de adesão às instruções fornecidas pelos profissionais de saúde; de fato, a comunicação eficaz entre os profissionais de saúde e as gestantes desempenha um papel central na adesão ao tratamento. As gestantes destacaram, ainda, a importância da atuação do enfermeiro na orientação sobre o uso correto dos antibióticos, o que revela a necessidade de instrumentos educativos para melhorar o atendimento e a saúde das usuárias e a relevância de ferramentas específicas para reforçar a prática clínica^(23)^.

No que diz respeito ao tempo de atuação como enfermeiro, mais da metade dos profissionais apresentou experiência sólida na APS, o que evidencia a maturidade técnica e a *expertise* acumuladas na prática clínica. Isso ressalta a importância de estratégias de desenvolvimento profissional contínuo para que os enfermeiros estejam capacitados a manejar condições complexas, como a ITU durante a gravidez^(24)^.

Em relação ao tempo de atuação no programa ESF, os dados refletiram um padrão semelhante, evidenciando a importância de uma trajetória profissional sólida nesse âmbito. A experiência acumulada na ESF é um fator determinante para a qualidade do cuidado, uma vez que esses profissionais desenvolvem habilidades específicas para a APS, incluindo a capacidade de realizar diagnósticos precoces e implementar intervenções preventivas no manejo da ITU. A continuidade da atuação nesse nível de atenção, associada à especialização em saúde coletiva, é fator determinante para a qualidade do cuidado prestado. Essa experiência é essencial, pois facilita o desenvolvimento de competências específicas. Ademais, a continuidade na APS permite maior familiaridade com os protocolos de atenção e maior vínculo com as gestantes^(25)^.

Outro dado relevante é que todos os enfermeiros avaliados haviam cursado pós-graduação, na maioria na área de saúde coletiva ou saúde pública. Esse perfil acadêmico é um diferencial, considerando que a APS demanda profissionais capacitados para abordar os determinantes sociais da saúde, além de promover estratégias de prevenção e cuidado direcionadas às gestantes^(25)^. Indica, assim, que um número expressivo de participantes se preocupou em aprimorar suas habilidades para se adaptar de maneira eficaz ao ambiente de trabalho, visto que a formação acadêmica e a prática cotidiana entrelaçam-se para possibilitar uma assistência humanizada e tecnicamente embasada. Espera-se que enfermeiros com especialização na área da saúde coletiva apresentem maior capacidade para integrar conhecimentos técnico-científicos às práticas de cuidado, resultando em maior adesão das gestantes aos tratamentos propostos^(26)^.

Por outro lado, as informações referentes à avaliação da percepção dos profissionais de saúde sobre a RAM e o manejo de antibióticos apontam lacunas significativas de formação e treinamento. Possivelmente, a insuficiência de educação continuada em saúde limita o protagonismo do enfermeiro com relação ao tema^(27,28)^. Em média, os profissionais também demonstraram insegurança para prescrever antibióticos para gestantes com ITU; no entanto, valores elevados foram registrados para a percepção da importância do treinamento em RAM, bem como a implementação de ações educativas na APS. Grande parte dos enfermeiros acredita que deve fazer parte do PGA na APS para o enfrentamento da RAM. Nesse sentido, há abertura para iniciativas de capacitação acerca do manejo do enfermeiro para prescrição de antibióticos para gestantes com ITU e inclusão de programas institucionais, como o PGA, para fortalecer o manejo da RAM^(9,10,29)^.

Este estudo apresentou algumas limitações, a exemplo da amostra pequena em uma única UBS, que não permitiu generalização; ressaltamos, porém, que este foi um estudo de caráter exploratório que deverá ser detalhado em etapas subsequentes. Outra limitação se referiu ao fato de não terem sido aprofundadas, em detalhes, as barreiras e facilitadores identificados pelos enfermeiros, o que será objeto de estudos futuros.

## CONCLUSÃO

A pesquisa realizada na UBS Parque Araribá, no município de São Paulo, trouxe à tona informações relevantes que podem contribuir para estratégias visando ao enfrentamento da RAM. Seus resultados permitem considerar que os objetivos norteadores da pesquisa foram alcançados, tendo sido possível identificar o conhecimento prévio sobre antibióticos das gestantes com ITU incluídas na pesquisa e a percepção dos enfermeiros sobre a existência de facilitadores e desafiadores que influenciam o cuidado a esse público.

Esperamos que os resultados contribuam para o aperfeiçoamento das práticas organizacionais, assistenciais e docentes, além de estimular reflexões sobre as políticas públicas. Recomendamos a validação e a implementação de tecnologia educativa com o público-alvo, por meio de novas pesquisas, a fim de garantir a eficácia da educação em saúde voltada a gestantes com ITU na APS.

Com base nos achados, a próxima fase deste trabalho será o desenvolvimento de uma cartilha de cuidado para os enfermeiros que atendem a gestantes com ITU, estratégia promissora para otimizar o tratamento e prevenir a RAM, alinhando as práticas de cuidado com as necessidades da população atendida. O produto técnico em formato de cartilha será disponibilizado no *site* do grupo de pesquisa Políticas Públicas, Epidemiologia e Tecnologias em Prevenção de Infecções Relacionadas à Assistência à Saúde (https://www.petiras.org/#).

Este artigo é um excerto da dissertação de mestrado, intitulada “O cuidado do enfermeiro na Atenção Primária À Saúde para enfrentamento da resistência antimicrobiana em gestantes com infecção do trato urinário”, defendida em 16 de abril de 2025 no Programa de Pós-Graduação Mestrado Profissional em Enfermagem na Atenção Primária à Saúde no Sistema Único de Saúde (MPAPS) da Escola de Enfermagem da Universidade de São Paulo.

## Data Availability

Todo o conjunto de dados que dá suporte aos resultados deste estudo está disponível mediante solicitação ao autor correspondente Jamile Leite de Figueiredo. O conjunto de dados não está publicamente disponível devido a conter informação que compromete a privacidade dos participantes da pesquisa.

# Translation: El cuidado del enfermero en la atención primaria de salud para hacer frente a la resistencia a los antimicrobianos en mujeres embarazadas con infección urinaria

**DOI:** 10.1590/1980-220X-REEUSP-2025-0001es

**Authors:** Jamile Leite de Figueiredo, Maria Clara Padoveze

**Affiliations:** 1 Universidade de São Paulo, Escola de Enfermagem, São Paulo, SP, Brasil.; 2 Einstein Hospital Israelita, São Paulo, SP, Brasil.

**Keywords:** Atención Primaria de Salud, Farmacorresistencia Microbiana, Personas Embarazadas, Infecciones Urinarias, Enfermeras de Familia

## Abstract

**Objetivo:**

Identificar a las mujeres embarazadas con infección del tracto urinario en seguimiento en una unidad básica de salud y sus conocimientos sobre los antibióticos, además de los factores facilitadores y desafiantes percibidos por los enfermeros que influyen en la atención, con especial atención a la resistencia a los antimicrobianos.

**Método:**

Estudio exploratorio, descriptivo, de enfoque cuantitativo, con mujeres embarazadas con infección del tracto urinario en tratamiento con antibióticos en una unidad del municipio de São Paulo y enfermeros del mismo lugar. Los datos, obtenidos a través de sistemas informáticos, historias clínicas y entrevistas, se sintetizaron y analizaron con los*programas informáticos* *Microsoft Excel*y*Stata*.

**Resultados:**

De las 21 gestantes incluidas, el 85,7 % tenía conocimientos sobre antibióticos. De los 11 enfermeros participantes, el 36,4 % no identificó la presencia de facilitadores, pero el 45,5 % creía que existían barreras en la atención.

**Consideraciones finales:**

La mayoría de las gestantes presentaban conocimientos básicos sobre los antibióticos y reconocían la importancia de la orientación de los enfermeros. Estos identificaron barreras que apuntan a la necesidad de apoyo técnico. Desarrollar un manual de cuidados para enfermeros que atienden a gestantes con infección del tracto urinario puede ser una estrategia prometedora para optimizar el tratamiento y prevenir la resistencia a los antimicrobianos.

## INTRODUCCIÓN

Las infecciones del tracto urinario (ITU) son respuestas inflamatorias causadas por la colonización e invasión de microorganismos en el sistema urinario, que afectan a cerca de 150 millones de personas en todo el mundo. Son especialmente comunes durante el embarazo, afectando entre el 5% y el 15% de las mujeres embarazadas, principalmente en el primer trimestre, con una prevalencia general de aproximadamente el 20%^(1–3)^. Pueden dar lugar a complicaciones maternas y fetales, y los diversos factores de riesgo asociados incluyen condiciones de salud, socioeconómicas y demográficas. Durante el embarazo, otros factores agravantes son los cambios anatómicos, fisiológicos y hormonales, como la compresión del útero y las alteraciones en los niveles hormonales, que favorecen la aparición de infecciones^(1)^.

En esta fase, las formas clínicas más comunes de ITU son la bacteriuria asintomática, seguida de la cistitis y la pielonefritis. La primera se caracteriza por un resultado de urocultivo de más de 100 000 UFC/ml de la misma bacteria, en cultivo de chorro medio de orina, sin signos ni síntomas. En otras palabras, se refiere a la presencia de bacterias en la orina en gran cantidad, pero sin repercutir en el desarrollo de manifestaciones clínicas^(1)^. Aunque no causa síntomas, su tratamiento es importante en mujeres embarazadas, ya que puede dar lugar a complicaciones graves, como la evolución hacia ITU sintomáticas, que pueden afectar a la salud de la madre y del feto. Hay otras complicaciones que pueden surgir como consecuencia de esta evolución, como pielonefritis, trombocitopenia, insuficiencia renal transitoria, limitación del crecimiento intrauterino, bajo peso del recién nacido (RN), muerte perinatal, rotura prematura de membranas amnióticas, aumento de la mortalidad fetal, prematuridad, parto prematuro y puerperio febril. Además, también aumenta la probabilidad de endometriosis posparto, eclampsia, anemias e hipertensión. El seguimiento y el tratamiento adecuados son esenciales para prevenir estos riesgos^(4)^.

Por el contrario, la ITU sintomática se acompaña de signos y síntomas clínicos, es decir, la principal diferencia entre la ITU sintomática y la bacteriuria asintomática radica en la presencia de síntomas y en la definición clínica de cada afección^(2)^. Entre las infecciones sintomáticas, la cistitis se produce en el tracto urinario inferior, afectando a la vejiga y la uretra, mientras que la pielonefritis se produce en el tracto urinario superior, cuando hay afectación renal. La cistitis es la forma sintomática más común y puede complicarse en personas con algún problema de salud que aumente el riesgo de fracaso del tratamiento. Entre las personas con mayor probabilidad de desarrollar complicaciones graves se encuentran las mujeres embarazadas, las personas que han sido sometidas a un trasplante de riñón, las que utilizan catéteres vesicales de demora, las que padecen patologías renales y enfermedades simultáneas, como diabetes mellitus e inmunosupresión^(4)^.

La posible aparición de resistencia a los antimicrobianos (RAM) es un factor importante a tener en cuenta, ya que hace que el tratamiento de las ITU en mujeres embarazadas sea aún más difícil. Actualmente, la RAM se considera uno de los principales retos en salud pública, como resultado de la manifestación de genes de resistencia que comprometen la eficacia de los antibióticos^(1)^, exacerbada por el uso inadecuado de antibióticos, como la automedicación y la dispensación sin receta, algunas de las principales causas del aumento de las tasas de resistencia^(1–4)^. Su diagnóstico precoz y su tratamiento adecuado son fundamentales para garantizar la salud de la gestante y del bebé, ya que una ITU no tratada puede provocar complicaciones^(3)^.

En Brasil, con el fin de contribuir a la lucha contra la RAM, la Agencia Nacional de Vigilancia Sanitaria (Anvisa) ha elaborado unas directrices para el Programa de Gestión de Antimicrobianos (PGA)^(5)^, un conjunto de medidas integradas que buscan garantizar el uso responsable de los antimicrobianos, siendo vital la participación de los enfermeros en este proceso^(6)^. El equipo de enfermería debe supervisar la administración de antimicrobianos y educar a los pacientes sobre su uso adecuado^(7,8)^.

En este contexto, la Atención Primaria de Salud (APS) desempeña un papel fundamental, ya que es responsable de atender una alta demanda de infecciones, incluidas las ITU, que son comunes durante el embarazo. Los enfermeros que trabajan en la APS desempeñan un papel esencial en la educación sobre el uso responsable de los antimicrobianos. Aunque un estudio ha indicado que el 74,1% de ellos desconocen el PGA, el 92,9% consideran importante la participación de la enfermería en estas acciones educativas^(9)^, siendo también fundamental la decisión compartida entre profesionales y usuarios para la gestión de la RAM. Entre otras actividades, la actuación de los enfermeros es crucial para garantizar que se realicen cultivos bacteriológicos antes de iniciar los antibióticos y para supervisar la adecuada adherencia al tratamiento, evitando la selección de microorganismos resistentes^(7)^.

El seguimiento prenatal realizado por enfermeras es indispensable para garantizar la atención de las gestantes, especialmente en lo que se refiere al manejo de las quejas urinarias, pero se sabe poco sobre la perspectiva de las enfermeras en relación con la ITU en gestantes, es decir, hay escasez de estudios nacionales que exploren este tema, lo que sugiere que las enfermeras deben involucrarse más en la cuestión. Además, la carga de trabajo puede ser una barrera para obtener conocimientos sobre el tema^(10)^.

Ante esto, el presente estudio tiene como objetivo identificar a las gestantes con infección del tracto urinario en seguimiento en una Unidad Básica de Salud (UBS) y sus conocimientos sobre antibióticos, además de los factores facilitadores y las barreras percibidos por los enfermeros que influyen en la atención, con enfoque en la resistencia a los antimicrobianos. Como parte de la estrategia para contribuir a la mejora de la atención, a partir de los resultados, pretendemos desarrollar un folleto de atención a las gestantes con ITU destinado a los enfermeros de la APS, con el objetivo de reducir la RAM.

## MÉTODO

### Diseño del Estudio

Se trata de un estudio exploratorio, descriptivo, de enfoque cuantitativo.

### Lugar

El estudio se llevó a cabo en la UBS Parque Araribá, situada en la Coordinación Sur, bajo la supervisión técnica de Salud de Campo Limpo, en el municipio de São Paulo, cuya administración corre actualmente a cargo del Hospital Israelita Albert Einstein. La unidad cuenta con diez equipos de la Estrategia de Salud Familiar (ESF), que atienden a unos 36 000 usuarios, con una agenda preestablecida, con citas diarias para la atención continuada programada y otras para el acceso por demanda espontánea^(11)^. Para la atención programada, como en el caso de la atención prenatal, las consultas se dividen según un protocolo de periodicidad: mensuales hasta la semana 28 de gestación, quincenales de la semana 28 a la 36, semanales de la semana 36 a la 40, alternando entre el enfermero y el médico^(12)^. En este servicio se realizan alrededor de 107 consultas prenatales al mes, según datos extraídos del Sistema Integrado de Gestión de la Asistencia Sanitaria (SIGA Salud) y Power BI - Portal de Gestión de Indicadores de la unidad en 2023.

### Población y Criterios de Selección

La recolección de datos se realizó en el período comprendido entre el 18 de julio y el 7 de noviembre de 2024, tras la aprobación del Comité de Ética en Investigación, teniendo como población las gestantes usuarias del UBS que recibieron prescripción de antibióticos para el tratamiento de ITU. Se consideraron preliminarmente elegibles todas las mujeres embarazadas de los diez equipos de la unidad, independientemente de su grupo etario, edad, sexo, número de gestaciones, paridad, número de hijos, raza/color, número de parejas, estado civil, nivel de escolaridad, ocupación, entre otros. La muestra estuvo compuesta por aproximadamente 25 usuarias, lo que corresponde al porcentaje máximo de incidencia del 20% descrito en la literatura^(13)^. El número se basó en datos de 2023, calculados a partir de la literatura y considerando la media de 184 embarazadas registradas en la UBS, según consta en el Programa Mãe Paulistana, un programa de la Prefectura de São Paulo de apoyo a la embarazada y al recién nacido. La selección se realizó mediante el análisis del historial de todas las gestantes atendidas en la unidad, evaluando tanto el historial físico como el Historial Clínico Electrónico del Ciudadano. Tras la comprobación de cada historial y la identificación de las gestantes que habían recibido prescripción de antibióticos para el tratamiento de la ITU, se invitó a las usuarias a participar. También participaron en el estudio enfermeros que prestaban asistencia en la unidad, cuya muestra estuvo formada por 11 profesionales, diez enfermeros del equipo y un responsable técnico.

En cuanto a los criterios de inclusión y exclusión, se incluyeron las gestantes con ITU en tratamiento con antibióticos atendidas en el UBS, las gestantes con infecciones recurrentes durante el período de gestación y los enfermeros que trabajaban en la unidad. Se excluyeron las gestantes procedentes de otras UBS por no haber datos suficientes para completar el cuestionario. En cuanto a los enfermeros participantes, se excluyeron aquellos que no prestaron servicio en la UBS durante el período de recolección por motivos de vacaciones o licencia. La primera investigadora de este estudio, prestadora de servicios en dicha unidad, también fue excluida.

### Recolección de Datos

En relación con las gestantes, las fuentes de información fueron: (i) Power BI de la Dirección de Atención Primaria y Redes Asistenciales del Hospital Israelita Albert Einstein, plataforma de análisis de datos para la generación de informes y*dashboards*, desarrollada por Microsoft, que permite a los usuarios visualizar y transformar datos de diversas fuentes; (ii) Plataforma SIGA Saúde: plataforma digital de gestión de los recursos y servicios prestados por la Secretaría Municipal de Salud de la Prefectura de São Paulo, que integra los procesos de asistencia, promoción y regulación de la salud en los establecimientos de la red municipal, en apoyo al Sistema Único de Saúde (SUS); (iii) PEC: modelo nacional de gestión de la información en la APS; (iv) historial de las gestantes, disponible en la UBS.

Tras identificar a las embarazadas con ITU, la investigadora las invitó a una reunión presencial, en la que les explicó los objetivos del estudio y los criterios de participación. En los casos en que la gestante no pudo concertar una cita presencial en la UBS, la investigadora realizó una búsqueda activa mediante visitas domiciliarias. Se invitó a los enfermeros que realizaban el seguimiento prenatal en la unidad a participar en el estudio para verificar sus conocimientos sobre la RAM y el uso de antibióticos, así como los factores facilitadores y desafiantes presentes en la atención.

Los datos se obtuvieron mediante cuestionarios específicos, uno para las gestantes con ITU y otro para los enfermeros, utilizando una escala Likert de cinco puntos (1-totalmente en desacuerdo; 2-parcialmente en desacuerdo; 3-neutro; 4-parcialmente de acuerdo; 5-totalmente de acuerdo) para medir el nivel de concordancia. Su elaboración se basó en la experiencia de las autoras, considerando la rutina de la asistencia prestada por los enfermeros a las gestantes en los servicios de APS, y en bibliografía de la área. Especialistas miembros del grupo de investigación revisaron los instrumentos para garantizar su alcance y aplicabilidad, los cuales fueron sometidos a una etapa piloto, con ajustes realizados según la necesidad antes del uso efectivo para la recolección de datos. Los cuestionarios están disponibles para su consulta poniéndose en contacto con la autora de la correspondencia.

El formulario para gestantes con ITU se organizó en cuatro partes: caracterización de la participante, destinada a los datos personales y el perfil de la paciente; datos del historial clínico, que se completó con los datos del historial, incluidos los datos gestacionales y obstétricos, información sobre la ITU adquirida, exámenes de laboratorio y prescripción de antibióticos que figuraban en el historial de salud; datos referidos por la paciente: relativos a las quejas de la gestante, así como sus síntomas y el momento en que se le recetó el antibiótico; conocimientos sobre los antibióticos: destinados a verificar los conocimientos previos de la gestante sobre los antibióticos y cómo realizó el tratamiento.

El formulario del enfermero se organizó en dos partes: perfil del enfermero, con información sobre su antigüedad como profesional y en la ESF, mejoras y títulos académicos; conocimientos del enfermero, incluyendo el conocimiento sobre el uso de antibióticos, RAM y PGA.

### Análisis y Tratamiento de los Datos

Los datos recopilados (tanto de los registros electrónicos como de los cuestionarios) se introdujeron en una hoja de cálculo, utilizando como herramienta de análisis los programas*Microsoft Excel*y*Stata*. Los resultados se analizaron mediante estadística descriptiva, distribución absoluta y relativa de las variables estudiadas y, cuando procedía, medida de tendencia central y dispersión, con el apoyo de un profesional de la estadística. Los datos se presentan en forma de gráficos y tablas, según corresponda.

### Aspectos Éticos

La investigación fue sometida al Comité de Ética en Investigación de la Escuela de Enfermería de la Universidad de São Paulo, a través de la Plataforma Brasil, de conformidad con la legislación vigente y mediante cartas de autorización de la dirección y supervisión de la Coordinación Sur de la Prefectura Municipal de São Paulo. Los datos del dictamen fundamentado del comité tienen el número 6.932.389 y el CAAE 78707224.1.0000.5392.

## RESULTADOS

El 1^o^ de agosto de 2024, tras la selección de las embarazadas elegibles para el estudio y la aplicación de los criterios de inclusión y exclusión, se invitó a las participantes a una consulta presencial a través de los equipos de la UBS. Al principio, hubo dificultades para que las pacientes se inscribieran, ya que los equipos no podían atender la demanda, al disponer de poco tiempo para contactar con ellas. La estrategia consistió en colocar recordatorios en los historiales y enviar mensajes a los profesionales médicos y enfermeros del grupo informativo de WhatsApp de la UBS solicitando que derivaran a las pacientes a la consulta con la investigadora. También se estableció contacto directo con las gestantes a través de las agentes comunitarias de salud, lo que permitió la recolección de datos entre septiembre y octubre.

Inicialmente se consideraron 29 participantes, de las cuales se excluyeron ocho (27,6%): dos se negaron, dos no fueron localizadas, dos se mudaron del territorio y dos faltaron a dos citas consecutivas y, tras ofrecérseles una visita domiciliaria, ambas la rechazaron. De las 21 (72,4%) restantes aptas para el análisis detallado, seis acudieron a consultas presenciales en el UBS y 15 recibieron visitas domiciliarias. La Tabla 1 muestra el perfil sociodemográfico de las gestantes con ITU.

**Tabla 1:** Perfil sociodemográfico de las gestantes con infección urinaria en la Unidad Básica de Salud Parque Araribá – São Paulo, SP, Brasil, 2024. N = 21.

Variable sociodemográfica	n	%
**Embarazo**		
Una	9	42,86
Dos	9	42,86
Tres o más	3	14,29
**Paridad**		
Parto normal	7	33,33
Aborto espontáneo	7	33,33
Aún no ha dado a luz	5	23,81
Cesárea	2	9,52
**Número de hijos vivos**		
Ninguno	12	57,14
Uno	6	28,57
Dos	1	4,76
Tres	1	4,76
Cinco	1	4,76
Cuatro	0	0,00
Número de compañeros*		
Sólo uno	20	95,24
Ninguno	1	4,76
**Antecedentes obstétricos**		
No	12	57,14
Sí	9	42,86
Aborto	7	33,33
Desprendimiento de la placenta	1	4,76
Hipertensión gestacional	1	4,76
**Riesgo gestacional**		
Habitual	17	80,95
Alto	4	19,05

Fuente: Elaboración propia.

*En el momento de la entrevista.

De las embarazadas, casi la mitad eran primigrávidas y algo menos de la mitad primíparas, mientras que una minoría tenía tres o más embarazos previos (multigrávida). En cuanto al tipo de parto, algo menos de la mitad declaró haber tenido un parto normal, mientras que una minoría había tenido una cesárea; el resto aún no había experimentado el parto. Alrededor de un tercio de las embarazadas declararon abortos espontáneos, algo más de la mitad no tenían hijos nacidos vivos y la mayoría declararon tener una sola pareja. La Tabla 2 muestra los datos de la ITU y otras pruebas realizadas, con sus respectivos resultados, así como una descripción de los antibióticos prescritos.

**Tabla 2:** Datos clínicos de infección urinaria, exámenes realizados con resultados y descripción de los antibióticos prescritos – São Paulo, SP, Brasil, 2024. N = 21.

Variable	n	%
**Datos de infección urinaria**		
Confirmada por pruebas de laboratorio	9	42,86
Confirmada por síntomas y pruebas de laboratorio	8	38,10
Confirmado por síntomas	4	19,05
**Orina 1**		
Alterado	19	90,48
Sin información	2	9,52
**Urocultura**		
Realizada	18	85,71
Positivo, *Escherichia coli*	10	47,62
Negativo	5	23,81
Positivo, sin otra información	1	4,76
Positivo, flora mixta, indicativo de contaminación	1	4,76
Positivo, *Klebsiella pneumoniae*	1	4,76
Sin información	2	9,52
No realizado	1	4,76
**Antibiograma**		
Realizado	10	47,62
Sensible a nitrofurantoína, cefalexina, bactrim, nitrofurantoína, fosfomicina	3	28,58
Resistencia a la ciprofloxacina, norfloxacina, ampicilina, fosfomicina, trimetoprim, sulfametoxazol	1	4,76
No realizado	9	42,86
Sin información	2	9,52
**Antibiótico prescrito**		
Cefalexina	10	47,62
Nitrofurantoína	8	38,10
Amoxicilina	1	4,76
Ceftriaxona	1	4,76
**Categoría del prescriptor**		
Médico de estrategia sanitaria familiar	16	76,19
Médico de otro servicio	4	19,05
Enfermera	1	4,76
**Diagnóstico**		
Sin información	12	57,14
Cistitis	2	9,52
Infección urinaria de localización no especificada	3	14,28
Bacteriuria asintomática	4	19,05
Otros problemas de salud*		
No	18	85,71
Sí	4	14,29
Diabetes	1	4,76
Anemia	1	4,76
Enfermedad aguda	2	9,52
**Uso de otros medicamentos durante el tratamiento**		
No	16	76,19
Sí	5	23,81
**Quejas actuales (después del tratamiento)**		
No	18	85,71
Sí	3	14,29
**Mejora después del tratamiento**		
Sí	18	85,71
No	3	14,29

Fuente: Elaboración propia.

*Las embarazadas pueden tener más de un problema de salud.

La mitad de las gestantes tuvieron la ITU confirmada exclusivamente por exámenes de laboratorio, mientras que poco menos de la mitad presentaron confirmación tanto por síntomas como por exámenes. En la mayoría de los casos, el análisis sumario de orina mostró alteraciones, y el urocultivo confirmó la presencia de*Escherichia coli*en concentraciones superiores a 100.000 UFC/mL en poco más de la mitad de los casos, siendo este el principal patógeno responsable de la ITU en gestantes^(6)^. En nuestra serie, solo un caso tuvo etiología por*Klebsiella pneumoniae.*A pesar de su importancia para orientar el tratamiento antimicrobiano, no se realizó un antibiograma en la otra mitad de los casos, lo que limitó la posibilidad de una terapia dirigida. Los datos indicaron que la mayoría de las gestantes utilizaron antibióticos en el segundo trimestre, seguido del primer y tercer trimestre. De las cuatro gestantes con bacteriuria asintomática confirmada, dos se encontraban en el primer trimestre de gestación (13 semanas y 9 semanas de gestación, respectivamente), y fueron identificadas en los exámenes de detección realizados durante la apertura del prenatal por el enfermero. En cuanto a la profilaxis, en la mayoría de los casos se prescribió cefalexina, seguida de nitrofurantoína. El diagnóstico no se detalló en la mayoría de los casos y el médico fue el principal prescriptor, en su mayoría procedente del ESF. Solo un enfermero fue identificado como prescriptor de metronidazol en crema. Este hallazgo puede deberse a un fallo en el registro, ya que la indicación diagnosticada no es compatible con el tratamiento.

De las 21 gestantes, cinco (23,8%) tuvieron recidiva de ITU, tres de ellas (14,3%) acudieron a consulta con el médico de la ESF y dos (52,0%) con médicos de otro servicio. En la recidiva, se mantuvo la elección de la cefalexina en tres casos (14,3%); en dos (9,5%), se utilizó fosfomicina. En cuanto al tiempo transcurrido entre las prescripciones, prevaleció el periodo de 7 a 14 días entre la lectura del examen y la prescripción de la medicación, seguido de 14 a 28 días. La mayoría de las evaluadas informaron síntomas relacionados con la ITU, como dolor en la parte baja del abdomen, disuria y polaquiuria, mientras que la minoría no presentó síntomas significativos, lo que puede indicar un subregistro de los casos asintomáticos o con molestias menores, posiblemente debido a las dificultades de las gestantes para expresar los síntomas. Solo dos no completaron el tratamiento hasta el final, una de las cuales tenía bacteriuria asintomática. La Tabla 3 presenta los datos relativos al conocimiento de las gestantes sobre los antibióticos.

**Tabla 3:** Distribución de respuestas de gestantes sobre conocimiento de antibióticos – São Paulo, SP, Brasil, 2024. N = 21.

Pregunta*	Totalmente en desacuerdo		Parcialmente en desacuerdo		Neutral		Parcialmente de acuerdo		Totalmente de acuerdo	
	n	%	n	%	n	%	n	%	n	%
¿Sabe qué son los antibióticos?	1	4,76	0	0,0	2	9,52	8	38,10	10	47,62
¿Ha utilizado alguna vez un antibiótico sin receta médica?	10	47,62	2	9,52	1	4,76	4	19,05	4	19,05
¿Suele tomar antibióticos en casa?	7	33,33	5	23,81	1	4,76	4	19,05	4	19,05
¿Ha utilizado los antibióticos que se dejó en casa?	12	57,14	2	9,52	4	19,05	1	4,76	2	9,52
¿Ha utilizado alguna vez los antibióticos que le dio un conocido?	13	61,91	2	9,52	0	0,0	3	14,29	3	14,29
¿Sabe para qué sirven los medicamentos recetados?	0	0,0	0	0,0	1	4,76	2	9,52	18	85,71
¿Olvidó tomar la medicación a la hora adecuada durante el tratamiento?	10	47,62	1	4,76	6	28,6	0,0%	28,6	4	19,05
¿Dejó de tomar la medicación en algún momento del tratamiento?	15	71,43	1	4,76	1	4,8	3	14,29	1	4,76
¿Le instruyó el profesional cuando le dio la medicación sobre su uso correcto y por qué?	1	4,76	0	0,0	0	0,0	1	4,76	19	90,48
Si recibió orientación, ¿quedó satisfecho con la información facilitada?	0	0,0	0	0,0	0	0,0	2	9,52	19	90,48
Cuando se le indica que lo utilice, ¿suele hacerlo tal y como se le indica?	0	0,0	0	0,0	0	0,0	3	14,29	18	85,71
¿Considera importante utilizar los antibióticos según las indicaciones?	0	0,0	0	0,0	0	0,0	2	9,52	19	90,48
En su opinión, ¿podría mejorarse el servicio prestado con respecto a la orientación sobre el uso de antibióticos?	2	9,52	1	4,76	1	4,76	6	28,6	11	52,38
En su opinión, ¿juega la enfermera un papel importante en los cuidados prestados en relación con la orientación sobre el uso de antibióticos?	0	0,0	0	0,0	0	0,0	3	14,29	18	85,71
¿Cree que es importante que las enfermeras dispongan de una herramienta para ayudar mejor a los pacientes en el uso de antibióticos?	1	4,76	0	0,0	0	0,0	3	14,3	17	80,95
¿Cree que esta herramienta puede mejorar la atención y, en consecuencia, la salud?	0	0,0	0	0,0	0	0,0	4	19,05	17	80,95

Fuente: Elaboración propia.

*Todas las preguntas fueron respondidas por las gestantes, no habiendo pérdidas de respuestas.

El análisis de los resultados del cuestionario reveló un alto nivel de acuerdo en las respuestas a la pregunta “¿Sabe lo que son los antibióticos?”, lo que indica un buen nivel de conocimientos básicos. El uso inadecuado de los antibióticos se mencionó en contadas ocasiones, aunque se notificaron prácticas inadecuadas, como el uso de antibióticos sin receta y de medicamentos sobrantes. La satisfacción con la orientación recibida fue alta, al igual que el nivel de cumplimiento de las instrucciones proporcionadas por los profesionales sanitarios. Las embarazadas destacaron la importancia del papel de la enfermera en la orientación sobre el uso correcto de los antibióticos.

La Tabla 4 muestra los resultados de la caracterización de las enfermeras participantes.

**Tabla 4:** Perfil de los enfermeros – São Paulo, SP, Brasil, 2024. N = 11.

Variable	n	%
**Edad**		
Entre 40 y 60 años	7	63,64
Hasta 39 años	3	27,27
Más de 60 años	1	9,09
**Antigüedad como enfermero de la Estrategia de Salud de la Familia**		
Entre 10 y 15 años	6	54,55
Hasta 9 años	3	27,27
Más de 15 años	2	18,18
**Siempre ha trabajado en Atención Primaria**		
Sí	8	72,73
No	3	27,27
**Tipo de institución universitaria**		
Privada	11	100,00
Otros	0	0,0
**Postgrado en Atención Primaria de Salud, salud pública o salud colectiva (finalizado)**		
Sí	9	81,82
No	2	18,18
**Estudios de postgrado en curso en otras áreas**		
No	10	90,91
Sí	1	9,09

Fuente: elaboración propia.

En cuanto al tiempo trabajando como enfermera, más de la mitad de las profesionales tenían entre 10 y 15 años de experiencia, lo que demuestra su madurez técnica y la *experiencia* acumulada en la práctica clínica. Algo menos de la mitad declararon tener hasta 9 años de experiencia y más de 15 años. En cuanto al tiempo de trabajo en la ESF, los datos reflejaron un patrón similar, con algo más de la mitad trabajando entre 10 y 15 años, mientras que la otra mitad se dividió entre hasta 9 y más de 15 años. La mayoría había trabajado siempre en APS, lo que demuestra una sólida trayectoria profesional en este ámbito. Otro dato relevante es que todos habían realizado un postgrado, especialmente en el área de salud pública o salud pública. Con relación a la continuidad de la formación, con perfeccionamiento en otra área, apenas uno (9,1%) informó haber realizado otro curso de postgrado. Otro (9,1%) había completado una residencia en su campo. Ninguna estaba estudiando o había realizado un doctorado. La evaluación de la percepción de los antibióticos por parte de los enfermeros se muestra en la Tabla 5.

**Tabla 5:** Distribución de las respuestas de los enfermeros sobre los antibióticos, la resistencia antimicrobiana y el Programa de Vigilancia Antimicrobiana – São Paulo, SP, Brasil, 2024. N = 11.

Pregunta*	Totalmente en desacuerdo		Parcialmente en desacuerdo		Neutral		Parcialmente de acuerdo		Totalmente de acuerdo	
	n	%	n	%	n	%	n	%	n	%
¿Sabe qué es la resistencia a los antimicrobianos?	1	9,09	0	0,0	0	0,0	2	18,2	8	72,73
¿Sabe qué es el Programa de Vigilancia Antimicrobiana y cuál es su finalidad?	3	27,3	2	18,2	1	9,09	4	36,36	1	9,09
¿Prescribe antibióticos establecidos por protocolos de enfermería del municipio de São Paulo o protocolos institucionales?	5	45,46	0	0,0	0	0,0	2	18,2	4	27,27
¿Se siente seguro recetando antibióticos?	4	36,36	1	9,09	1	9,09	2	18,2	3	27,27
¿Cree que tiene suficientes conocimientos para prescribirlos?	3	27,27	1	9,09	2	18,2	3	27,3	2	18,2
¿Se siente seguro recetando antibióticos a mujeres embarazadas con infecciones urinarias?	4	36,36	1	9,09	2	18,2	2	18,2	2	18,2
¿Aconseja a los pacientes sobre el uso correcto de los antibióticos y por qué?	0	0,0	0	0,0	0	0,0	2	18,2	9	81,82
¿Sabe qué precauciones deben tomarse con los pacientes cuando se les prescribe un antibiótico para reducir la resistencia a los antimicrobianos?	0	0,0	0	0,0	0	0,0	4	36,36	7	63,64
¿Conoce la gestión de la resistencia a los antimicrobianos?	2	18,2	4	36,36	1	9,09	3	27,3	1	9,09
¿Ha recibido algún tipo de formación sobre la resistencia a los antimicrobianos y su gestión?	6	54,55	1	9,09	2	18,2	1	9,09	1	9,09
En su opinión, ¿cree que es importante recibir formación sobre la resistencia a los antimicrobianos y su gestión en Atención Primaria?	0	0,0	0	0,0	0	0,0	0	0,0	11	100,0
¿Ha participado alguna vez en actividades educativas sobre la resistencia a los antimicrobianos?	8	72,73	1	9,09	0	0,0	2	18,2	0	0,0
En su opinión, ¿cree que es importante realizar actividades educativas en Atención Primaria para la comunidad que aborden la resistencia a los antimicrobianos?	0	0,0	0	0,0	0	0,0	1	9,09	10	90,91
En su opinión, ¿cree que es importante contar con el Programa de Vigilancia Antimicrobiana en Atención Primaria?	0	0,0	0	0,0	1	9,09	1	9,09	9	81,82
¿Cree que las enfermeras deberían formar parte del Programa de Vigilancia Antimicrobiana en Atención Primaria para hacer frente a la resistencia antimicrobiana?	0	0,0	0	0,0	1	9,09	1	9,09	9	81,82
¿Le gustaría disponer de una herramienta que pueda orientarle para contribuir a los cuidados en relación con el uso de antibióticos y la gestión de la resistencia a los antimicrobianos?	0	0,0	0	0,0	0	0,0	3	27,3	8	72,73
¿Cree que esta herramienta puede mejorar su atención en relación con el uso de antibióticos?	0	0,0	0	0,0	0	0,0	2	18,2	9	81,82
¿Cree que esta herramienta contribuirá a reducir la resistencia a los antimicrobianos en Atención Primaria?	0	0,0	0	0,0	1	9,09	1	9,09	9	81,82
En su opinión, ¿existe algún obstáculo en su asistencia que influya en su forma de abordar la resistencia a los antimicrobianos?	0	0,0	0	0,0	3	27,3	3	27,3	5	45,46
En su opinión, ¿existen facilitadores en su asistencia que influyan en la atención a la resistencia a los antimicrobianos?	4	36,36	0	0,0	2	18,2	3	27,3	2	18,2

Fuente: Elaboración propia.

*Todas las preguntas fueron contestadas por las enfermeras, por lo que no hubo pérdida de respuestas.

Los resultados mostraron que la mayoría de los profesionales tienen conocimientos básicos sobre la RAM, con un nivel aceptable de respuestas a la pregunta «¿Sabe qué es la resistencia a los antimicrobianos?». Sin embargo, en la pregunta «¿Sabe qué es el PGA y cuál es su objetivo?», casi la mitad de los enfermeros respondieron que no lo sabían, mientras que, en la pregunta «¿Ha recibido alguna formación sobre la RAM y su manejo?», poco más de la mitad respondieron que no. Casi la mitad de los profesionales también están totalmente o parcialmente en desacuerdo con que se sienten seguros para recetar antibióticos a mujeres embarazadas con ITU (n = 5; 45,5%). En cuanto a la percepción de la importancia de la formación en RAM, todos atribuyeron un alto grado de relevancia a la cuestión, así como a la implementación de acciones educativas en la APS. En relación con el PGA, la mayoría consideró que el programa debe implantarse en la APS, ya que creen que el enfermero debe formar parte de él en este ámbito para hacer frente a la RAM.

Entre los enfermeros entrevistados, cinco (45,5%) estaban totalmente de acuerdo en que existen barreras en la atención que influyen en los cuidados para hacer frente a la RAM. Por el contrario, solo dos (18,2%) estaban totalmente de acuerdo con la existencia de facilitadores en esta atención. En cuanto a las particularidades de estas barreras y facilitadores, no fue posible obtener información más detallada debido a las limitaciones del método, lo que requiere una exploración más detallada en futuros estudios.

## DISCUSIÓN

El perfil sociodemográfico de las embarazadas con ITU evidenció la influencia de factores como la edad, la etnia, el estado civil, la escolaridad y la ubicación geográfica, que pueden estar asociados a desigualdades en el acceso a la atención sanitaria e impactar en el manejo y la prevención de la ITU. Esta información es esencial para la implementación de intervenciones específicas y eficaces^(4,14)^, ya que la migración y el acceso desigual a los servicios de salud pueden contribuir a la prevalencia de la ITU en determinadas poblaciones^(14)^.

La combinación de orina 1, urocultivo y antibiograma proporciona un enfoque diagnóstico completo y eficaz para el manejo de la ITU en mujeres embarazadas, permitiendo identificar los agentes etiológicos, determinar la sensibilidad a los antimicrobianos y personalizar el tratamiento. El uso de exámenes como el urocultivo y el antibiograma para confirmar la presencia de patógenos y la sensibilidad a los antimicrobianos es esencial para elegir el antibiótico correcto. Este enfoque no solo mejora los resultados clínicos, sino que también reduce la incidencia de RAM, especialmente en contextos en los que son frecuentes las bacterias multirresistentes^(15)^. Sin embargo, en nuestra serie de casos observamos que no siempre se realizó el antibiograma.

Otro factor importante es la realización oportuna de estos exámenes de laboratorio, ya que es fundamental para la detección y confirmación de las ITU. Los diagnósticos precoces permiten intervenciones rápidas, reduciendo el riesgo de complicaciones, como pielonefritis y parto prematuro; los estudios indican que los retrasos en el diagnóstico están asociados con una mayor morbilidad en mujeres embarazadas y recién nacidos^(16)^. Por lo tanto, el tiempo transcurrido entre la realización de los exámenes diagnósticos y la prescripción del tratamiento es un factor determinante en el manejo clínico de las ITU en embarazadas^(1)^, ya que puede impactar directamente en la progresión de la infección y en los resultados materno-fetales, especialmente debido a los cambios fisiológicos del embarazo que aumentan la susceptibilidad a complicaciones^(17)^. Por otra parte, los antibióticos recetados en función del momento del embarazo, la elección del tipo de antibiótico y el momento de su administración deben analizarse cuidadosamente debido a sus posibles repercusiones en la salud materno-fetal^(18)^. En este estudio, se observó la presencia de bacteriuria asintomática en el primer trimestre, lo que indica la necesidad de que el personal de enfermería de la ESF preste atención al cribado precoz de la infección.

En cuanto al tratamiento farmacológico, el estudio demostró un manejo adecuado y la elección de antibióticos de baja toxicidad y riesgo para este grupo^(2,4)^. Sin embargo, la mayoría de los diagnósticos no fueron declarados, lo que puede atribuirse a la falta de registros adecuados en los historiales. La ausencia de información detallada en el historial médico puede comprometer el diagnóstico precoz y el manejo adecuado de las ITU^(19)^. De acuerdo con los resultados obtenidos, la mayoría de los prescriptores pertenecen a la categoría médica, procedentes de la ESF, lo que apunta a la necesidad de una mayor interacción entre el enfermero de la UBS y el profesional prescriptor para garantizar una atención más integrada a la gestante.

La aplicación de cuestionarios con escala Likert ha demostrado ser una herramienta eficaz para evaluar la comprensión y las percepciones de las gestantes sobre la ITU, ya que permite identificar de forma estandarizada actitudes, creencias y niveles de conocimiento, facilitando el análisis de datos cualitativos y cuantitativos. Los resultados de este estudio demostraron que, aunque existe un buen nivel de conocimiento y adherencia al uso de antibióticos entre las gestantes, aún se producen prácticas inadecuadas^(20)^que reflejan lagunas en la comprensión del uso responsable de los antibióticos^(21)^. Este conocimiento desempeña un papel fundamental en la adherencia al tratamiento y en la reducción de las complicaciones relacionadas con la ITU; a su vez, el uso subóptimo o inadecuado de los antibióticos puede indicar fallos en la adherencia al tratamiento. Además, la falta de adherencia puede estar asociada a barreras socioeconómicas, falta de conocimiento sobre los riesgos de las infecciones no tratadas y reticencia a utilizar medicamentos durante el embarazo^(21,22)^. La actuación de los enfermeros y el uso de instrumentos educativos personalizados son estrategias para mejorar la gestión del uso de antibióticos y prevenir la RAM.

La satisfacción con las orientaciones recibidas fue alta, al igual que el nivel de adherencia a las instrucciones proporcionadas por los profesionales de la salud; de hecho, la comunicación eficaz entre los profesionales de la salud y las mujeres embarazadas desempeña un papel fundamental en la adherencia al tratamiento. Las gestantes también destacaron la importancia de la actuación del enfermero en la orientación sobre el uso correcto de los antibióticos, lo que revela la necesidad de instrumentos educativos para mejorar la atención y la salud de las usuarias y la relevancia de herramientas específicas para reforzar la práctica clínica^(23)^.

En cuanto al tiempo de ejercicio como enfermero, más de la mitad de los profesionales presentaban una sólida experiencia en APS, lo que evidencia la madurez técnica y la*experiencia*acumulada en la práctica clínica. Esto resalta la importancia de estrategias de desarrollo profesional continuo para que los enfermeros estén capacitados para manejar afecciones complejas, como la ITU durante el embarazo^(24)^.

En relación con el tiempo de actuación en el programa ESF, los datos reflejaron un patrón similar, lo que evidencia la importancia de una trayectoria profesional sólida en este ámbito. La experiencia acumulada en el ESF es un factor determinante para la calidad de la atención, ya que estos profesionales desarrollan habilidades específicas para la APS, incluida la capacidad de realizar diagnósticos precoces e implementar intervenciones preventivas en el manejo de la ITU. La continuidad de la actuación en este nivel de atención, asociada a la especialización en salud colectiva, es un factor determinante para la calidad de la atención prestada. Esta experiencia es esencial, ya que facilita el desarrollo de competencias específicas. Además, la continuidad en la APS permite una mayor familiaridad con los protocolos de atención y un mayor vínculo con las gestantes^(25)^.

Otro dato relevante es que todos los enfermeros evaluados habían cursado estudios de posgrado, en su mayoría en el área de salud colectiva o salud pública. Este perfil académico es un diferencial, considerando que la APS exige profesionales capacitados para abordar los determinantes sociales de la salud, además de promover estrategias de prevención y atención dirigidas a las gestantes^(25)^. Esto indica que un número significativo de participantes se preocupó por mejorar sus habilidades para adaptarse de manera eficaz al entorno laboral, ya que la formación académica y la práctica cotidiana se entrelazan para permitir una asistencia humanizada y con base técnica. Se espera que los enfermeros con especialización en el área de la salud colectiva presenten una mayor capacidad para integrar los conocimientos técnico-científicos a las prácticas de cuidado, lo que se traduce en una mayor adherencia de las gestantes a los tratamientos propuestos^(26)^.

Por otro lado, la información relativa a la evaluación de la percepción de los profesionales de la salud sobre la RAM y el manejo de los antibióticos apunta a importantes lagunas en la formación y el entrenamiento. Posiblemente, la insuficiencia de la educación continua en salud limita el protagonismo del enfermero en relación con el tema^(27,28)^. En promedio, los profesionales también mostraron inseguridad para prescribir antibióticos a mujeres embarazadas con ITU; sin embargo, se registraron valores elevados en cuanto a la percepción de la importancia de la formación en RAM, así como la implementación de acciones educativas en la APS. La mayoría de los enfermeros cree que debe formar parte del PGA en la APS para hacer frente a la RAM. En este sentido, hay apertura para iniciativas de capacitación sobre el manejo del enfermero para la prescripción de antibióticos a gestantes con ITU y la inclusión de programas institucionales, como el PGA, para fortalecer el manejo de la RAM^(9,10,29)^.

Este estudio presentó algunas limitaciones, como el tamaño reducido de la muestra en una única UBS, lo que no permitió la generalización; sin embargo, cabe destacar que se trata de un estudio de carácter exploratorio que deberá detallarse en etapas posteriores. Otra limitación se refiere al hecho de que no se profundizó en detalle en las barreras y facilitadores identificados por los enfermeros, lo que será objeto de estudios futuros.

## CONCLUSIÓN

La investigación realizada en la UBS Parque Araribá, en el municipio de São Paulo, ha puesto de manifiesto información relevante que puede contribuir a las estrategias destinadas a hacer frente a la RAM. Sus resultados permiten considerar que se alcanzaron los objetivos que guiaron la investigación, ya que fue posible identificar los conocimientos previos sobre antibióticos de las gestantes con ITU incluidas en la investigación y la percepción de los enfermeros sobre la existencia de facilitadores y desafiadores que influyen en la atención a este público.

Esperamos que los resultados contribuyan a mejorar las prácticas organizativas, asistenciales y docentes, además de estimular la reflexión sobre las políticas públicas. Recomendamos la validación y la implementación de tecnología educativa con el público objetivo, mediante nuevas investigaciones, con el fin de garantizar la eficacia de la educación en salud dirigida a las gestantes con ITU en la APS.

Basándonos en los resultados, la siguiente fase de este trabajo será el desarrollo de un manual de cuidados para enfermeras que atienden a mujeres embarazadas con ITU, una estrategia prometedora para optimizar el tratamiento y prevenir la RAM, alineando las prácticas de cuidados con las necesidades de la población atendida. El producto técnico en formato de folleto estará disponible en el*sitio web*del grupo de investigación Políticas Públicas, Epidemiología y Tecnologías en Prevención de Infecciones Relacionadas con la Atención Sanitaria (https://www.petiras.org/#).

Este artículo es un extracto de la disertación de maestría, titulada “El cuidado del enfermero en la Atención Primaria de Salud para enfrentar la resistencia antimicrobiana en gestantes con infección del tracto urinario”, defendida el 16 de abril de 2025 en el Programa de Posgrado de Maestría Profesional en Enfermería en la Atención Primaria a la Salud en el Sistema Único de Salud (MPAPS) de la Escuela de Enfermería de la Universidad de São Paulo.

## Data Availability

Todo el conjunto de datos que apoya los resultados de este estudio está disponible a solicitud del autor correspondiente Jamile Leite de Figueiredo. El conjunto de datos no está disponible públicamente debido a que contiene información que compromete la privacidad de los participantes de la investigación.
